# Research advances in the *Pyrenophora teres–*barley interaction

**DOI:** 10.1111/mpp.12896

**Published:** 2019-12-13

**Authors:** Shaun J. Clare, Nathan A. Wyatt, Robert S. Brueggeman, Timothy L. Friesen

**Affiliations:** ^1^ Department of Plant Pathology North Dakota State University Fargo ND 58108‐6050 USA; ^2^ USDA‐ARS Cereal Crops Research Unit Northern Crop Science Laboratory Edward T. Schafer Agricultural Research Center 1616 Albrecht Boulevard N Fargo ND 58102‐2765 USA; ^3^ Present address: Department of Crop and Soil Science Washington State University Pullman WA 99164‐6420

**Keywords:** barley, effector, necrotrophic fungus, *Pyrenophora teres* f. *maculata*, *Pyrenophora teres* f. *teres*, resistance, susceptibility

## Abstract

*Pyrenophora teres* f. *teres* and *P. teres* f. *maculata* are significant pathogens that cause net blotch of barley. An increased number of loci involved in *P. teres* resistance or susceptibility responses of barley as well as interacting *P. teres* virulence effector loci have recently been identified through biparental and association mapping studies of both the pathogen and host. Characterization of the resistance/susceptibility loci in the host and the interacting effector loci in the pathogen will provide a path for targeted gene validation for better‐informed release of resistant barley cultivars. This review assembles concise consensus maps for all loci published for both the host and pathogen, providing a useful resource for the community to be used in pathogen characterization and barley breeding for resistance to both forms of *P. teres*.

## Graphical abstract

*Pyrenophora teres* causes net blotch of barley. Here we review the latest progress on host resistance and pathogen virulence, providing a valuable resource for the barley net blotch research community.

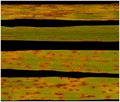

## Introduction

*Pyrenophora teres* is the causal agent of net blotch of barley and is found in two forms, *P. teres* f. *teres* and *P. teres* f. *maculata*, the causal agents of net form net blotch (NFNB) and spot form net blotch (SFNB), respectively (Smedegård‐Petersen, 1971). Net blotch is one of the most economically important diseases of barley, with typical yield losses ranging from 10% to 40% and possible complete crop failure in extreme cases (Galano *et al.*, 2011; Jayasena *et al.*, 2007; Jebbouj and El Yousfi, 2009; Khan, 1987; Moya *et al.*, 2018; Piening and Kaufmann, 1969). Net blotch is present in all barley‐growing regions of the world (Shipton *et al.*, 1973), including northern (Douiyssi *et al.*, 1998), eastern (Zeleke, 2017) and southern Africa (Louw *et al.*, 1995), Middle (Arabi *et al.*, 2003) and Far‐East Asia (Sato and Takeda, 1997), Europe (Arabi *et al.*, 1992), North (Tekauz, 1990) and South America (Moya *et al.*, 2018), and Oceania (Cromey and Parkes, 2003; Williams *et al*., 1999). The potential economic loss to *P. teres* f. *teres* and *P. teres* f. *maculata* has been valued at approximately A$117 and A$192 million per annum, respectively, in Australia alone (Murray and Brennan, 2010).

Originally named *Helminthosporium teres* [Sacc.] in 1809, the fungus was renamed *P. teres* Drechs. (anamorph *Drechsler teres* [Sacc.] Shoem.) in 1930 (Shoemaker, 1959). *P. teres* was subsequently divided into the two forms *P. teres* f. *teres* and *P. teres* f. *maculata* by Smedegård‐Petersen (1971) based on the lesion types. *P. teres* f. *teres* develops necrotic lesions with distinct striations, developing the net‐like pattern for which it was named. *P. teres* f. *maculata* develops oval necrotic lesions with a chlorotic halo (Shipton *et al.*, 1973; Smedegård‐Petersen, 1971). Arguably, these two forms could be classified as separate species based on their genetic isolation (Ellwood *et al.*, 2012; Jalli, 2011; Rau *et al.*, 2007; Syme *et al.*, 2018) and the rarity of hybridization of the two forms in nature, despite coinhabiting the same fields (Akhavan *et al.*, 2015; Campbell *et al.*, 2002; Çelik Oğuz *et al.*, 2018; Leišova *et al.*, 2005; McLean *et al.*, 2014; Poudel *et al.*, 2019). Hybrids can be induced in the lab (Campbell and Crous, 2003), suggesting a natural barrier to fertility or a fitness penalty in nature (Syme *et al.*, 2018). The two forms are reported to have diverged from each other 519 kya (±30) (Ellwood *et al.*, 2012; Syme *et al.*, 2018), and from one of their closest relatives *Pyrenophora tritici‐repentis,* the causal agent of tan spot on wheat 8.04 Mya (±138 kya) (Ellwood *et al.*, 2012; Ohm *et al.*, 2012).

One *P. teres* form is often dominant within a geographical area. *P. teres* f. *maculata* has become more prevalent in North Dakota (Liu and Friesen, 2010) and Idaho, USA (Marshall *et al.*, 2015) and Victoria, Australia (McLean *et al.*, 2010a), but this likely depends on the susceptibility of the predominant cultivars planted in each region. Multiple differential barley sets have been proposed to classify global pathotypes of *P. teres* f. *teres* (Afanasenko *et al.*, 2009, 1995; Fowler *et al.*, 2017) and *P. teres* f*. maculata* isolates in local populations (McLean *et al.*, 2014, 2010b). The taxonomy, population diversity, symptoms, life cycle, infection cycle and control of, and preliminary insights into the *P. teres–*barley interaction were previously reviewed by Liu *et al. *(2011), and therefore these aspects will not be covered here. In this review, we have collated the recent literature that lays the foundation for the functional characterization of *P. teres–*barley interactions.

### Genetics of barley reactions to *P. teres* f. *teres*

**Figure 1:**
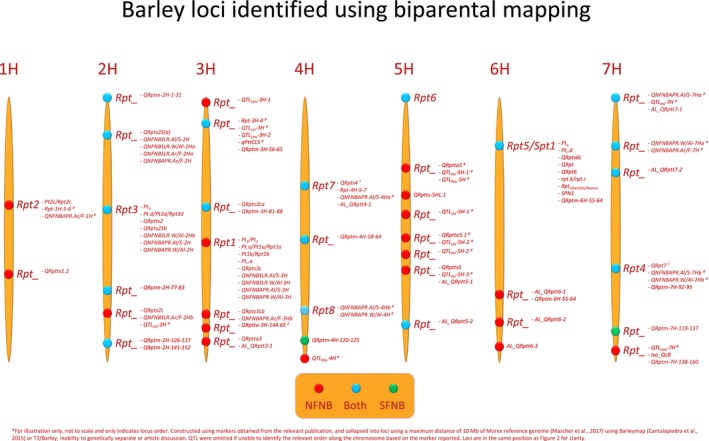
Consensus map of loci that are associated with net form net blotch (NFNB, red), spot form net blotch (SFNB, green) or both (blue) resistance/susceptibility across the barley genome identified using biparental mapping. Barley chromosome numbers are indicated at the top of each chromosome in sequential order. Designated loci are indicated by sequential numbering whereas previously undesignated loci are marked by an underscore (see also Table S3).

Resistance to *P. teres* f. *teres* was first shown to be quantitatively inherited by Geschele (1928). Schaller and Wiebe (1952) screened barley lines for resistance to *P. teres*, but it is unknown which form was used, concluding that lines, primarily of Manchurian origin, contained enough resistance for introgression into elite barley lines. The first resistance locus implicated in the *P. teres–*barley interaction was harboured by Tifang in a Tifang × Atlas cross, inherited as a single gene trait and effective against Californian *P. teres* isolates (Schaller, 1955). Mode and Schaller (1958) subsequently designated this locus *Pt1* and identified two additional loci, designated *Pt2* and *Pt3* (Table 1). The lines Ming, Manchurian and Harbin harboured *Pt2* as a single locus, whereas the lines Canadian Lake Shore and CI4922 carried *Pt2* and *Pt3* (Mode and Schaller, 1958)*.* The *Pt1* and *Pt2* loci were shown to be closely linked with 2.57% recombination by using resistant by resistant crosses (Mode and Schaller, 1958). An additional locus, designated *Pt_a_*, was discovered conferring resistance to the Australian isolate W.A.‐2 that was distinct from *Pt1, Pt2* and *Pt3* (Khan and Boyd, 1969a). Khan and Boyd (1969a) concluded that the genetic makeup of resistance in Harbin, Manchuria, Tifang and Ming was different depending on the isolate used based on the existing research at the time. Following the trisomic analysis of Bockelman *et al. *(1977) that failed to separate *Pt1* and *Pt2,* and the revision of the barley gene nomenclature, the *Pt1* and *Pt2* loci were collapsed into the *Rpt1* locus on the long arm of chromosome 3H with *Rpt1.a* and *Rpt1.b* alleles from Tifang/Harbin and CI9819, respectively (Table 1). Additional subsequent loci that were collapsed into the *Rpt1* locus included *Pta*, *QRpts3L* and *QNFNBSLR.Al/S‐3H* from Igri (Graner *et al.*, 1996), Arapiles (Raman *et al.*, 2003) and Alexis (Lehmensiek *et al.*, 2007), respectively (Table 1). The chromosome 3H resistance locus in Canadian Lake Shore mapped to the short arm of chromosome 3H (Dinglasan *et al.*, 2019), indicating that the Canadian Lake Shore resistance is not *Rpt1* (Fig. 1). Confusion over the *Pt2* locus occurred when *Rpt2.c* reported by Bockelman *et al. *(1977) was assumed to be *Pt2* reported by Mode and Schaller (1958). However, *Rpt2.c* in CI9819 is distinct from *Pt2,* which is allelic to other sources of resistance that map to the *Rpt1* locus (Bockelman *et al.*, 1977) (Table 1). Manninen *et al. *(2006) reported a major and a minor effect locus effective against different *P. teres* f. *teres* isolates on chromosomes 6H and 1H, respectively, in a Rolfi × CI9819 population, corroborating *Rpt2.c* on chromosome 1H (Table 1).

**Table 1:** Summary of current designated barley genes defined in the *Pyrenophora teres* f. *teres* and *P. teres* f. *maculata–*barley pathosystem including the current and previous synonyms, known alleles, barley chromosome and form of *P. teres* effective against*.* Different loci are indicated by alternating grey scale.

Designated locus (ref) *	Current synonym (ref) *	Previous synonyms (ref) *	Known alleles	Location	Form‡
*Rpt1* (Bockelman *et al*., 1977)		*Pt_1_* (Mode and Schafer, 1958) *Pt_2_* (Mode and Schafer, 1958) *Pt1a/Rpt1a* (Bockelman *et al*., 1977) *Pt1b/Rpt1b* (Bockelman *et al*., 1977) *Pt,,a* (Graner *et al*., 1996) *QRpts3L* (Raman *et al*., 2003) *QNFNBSLR.Al/S‐3H* (Lehmensiek *et al*., 2007) *QNFNBSLR.W/Al‐3H* (Lehmensiek *et al*., 2007) *QNFNBAPR.Al/S‐3H* (Lehmensiek *et al*., 2007) *QNFNBAPR.W/Al‐3H* (Lehmensiek *et al*., 2007)	*Rpt1.a* *Rpt1.b*	3H	*P. teres* f. *teres*
*Rpt2* (Bockelman *et al*., 1977)		*Pt2c/Rpt2c* (Bockelman *et al*., 1977) *Rpt‐1H‐5‐6* *#* (Yun *et al*., 2005) *QNFNBAPR.Ar/F‐1H* *#* (Lehmensiek *et al*., 2007)	*Rpt2.c*	1H	*P. teres* f. *teres*
*Rpt3* (Bockelman *et al*., 1977)		*Pt_3_* (Mode and Schaller, 1958) *Pt.d/Pt3d/Rpt3d* (Bockelman *et al*., 1977) *QRpts2S (*Raman *et al*., 2003 *)* *QRpts2Sa* (Raman *et al*., 2003) *QRptts2* (Grewal *et al*., 2008) *QNFNBSLR.Al/S‐2H* (Lehmensiek *et al*., 2007) *QNFNBSLR.W/Al‐2H* (Lehmensiek *et al*., 2007) *QNFNBSLR.Ar/F‐2Ha* (Lehmensiek *et al*., 2007) *QNFNBAPR.Ar/F‐2H* (Lehmensiek *et al*., 2007) *QPt.2H‐1/2* (Vatter *et al*., 2017)	*Rpt3.d*	2H	***P. teres* f. *teres*** *P. teres* f. *maculata*
*Rpt4* (Williams *et al*., 1999, 2003)		*QRpt7* (Grewal *et al*., 2008) *QNFNBAPR.Al/S‐7Hb* # (Lehmensiek *et al*., 2007) *QNFNBAPR.W/Al‐7Hb* # (Lehmensiek *et al*., 2007) *QRpt7* (Tamang *et al*., 2015) *QRptm7‐3/4/5/6/7/8* (Wang *et al*., 2015) *NBP_QRPtt7‐2* (Wonneberger *et al*., 2017b) *Qns‐7H.2* (Daba *et al*., 2019) *QRptm*‐*7H*‐*119*‐*137 (*Tamang *et al*., 2019 *)*	*Rpt4.e*	7H	*P. teres* f. *teres* ***P. teres* f. *maculata***
*Rpt5* (Manninen *et al*., 2006)	*Spt1* (Richards *et al*., 2016)	*Pt_a_* (Khan and Boyd, 1969a) *Pt,,d* (Graner *et al*., 1996) *QRpts6L* (Raman *et al*., 2003) *QRpt *(Embiri *et al*., 2005) *rpt.r/rpt.r* (Abu Qamar *et al*., 2008) *QRpt6* (Grewal *et al*., 2008, 2012) *Rpt_Ciho2291/Nomini_* (O’Boyle *et al*., 2014) *SPN1* (Liu *et al*., 2015) *QRptm6‐2* (Wang *et al*., 2015) *QPt.6H‐1/2* (Vatter *et al*., 2017) *NBP_QRPtt6‐1* (Wonneberger *et al*., 2017b) *QRptta‐6H‐49.79* (Amezrou *et al*., 2018) *QRptt.6H‐54‐55* (Amezrou *et al*., 2018) *Qns‐6H.5* (Daba *et al*., 2019) *Qnfnb‐6H.1‐4* (Daba *et al*., 2019) *Qsfnb‐6H* (Daba *et al*., 2019) *QRptm‐6H‐55‐64* (Tamang *et al*., 2019)	*Rpt5.f* *Spt1.k* *Spt1.r*	6H	***P. teres* f. *teres*** *P. teres* f. *maculata*
*Rpt6* (Manninen *et al*., 2006)		‐	*Rpt6.g*	5H	*P. teres* f. *teres* ***P. teres* f. *maculata***
*Rpt7* (Franckowiak and Platz, 2013)		*QRpts4* (Raman *et al*., 2003) † *Rpt‐4H‐5‐7* (Yun *et al*., 2005) *QNFNBAPR.Al/S‐4Ha* *#* (Lehmensiek *et al*., 2007) *AL_QRptt4‐1* (Wonneberger *et al*., 2017a) *QPt.4H‐3* # (Vatter *et al*., 2017) *QRptm‐4H‐58‐64* (Tamang *et al*., 2019)	*Rpt7.h*	4HL	***P. teres* f. *teres*** *P. teres* f. *maculata*
*Rpt8* (Franckowiak and Platz, 2013)		QTL on 4H (Friesen *et al*., 2006) *QRpts4* # (Grewal *et al*., 2008) † *Qns‐4H.2* (Daba *et al*., 2019)	*Rpt8.j*	4HS	***P. teres* f. *maculata***

* First paper to use gene designation.

^#^ Not confirmed.

^†^ Multiple papers use this designation.

^‡^ Bold indicates predominant form resistance is associated with.

The *Rpt3.d* locus located on chromosome 2H (Graner *et al.*, 1996) is commonly used as a synonym for the *Pt3/Pt.d* locus identified by Mode and Schaller (1958) based on the Bockelman *et al. *(1977) reclassification in Tennessee Awnless D22‐5 (Franckowiak and Platz, 2013) (Table 1). The *Rpt3.d* locus also includes *QRptts2* from TR251 (Grewal *et al.*, 2012) (Table 1). Other reported loci identified on chromosome 2H that may be part of the *Rpt3* locus include quantitative trait loci (QTLs) in Steptoe (Steffenson *et al.*, 1996), Kaputar (Cakir *et al.*, 2003), Franklin (Raman *et al.*, 2003) and ND11213 (Emebiri *et al.*, 2005) (Table S1).

The *Pt_a_* locus was reclassified as *Rpt5* on chromosome 6H with at least three alleles (Franckowiak and Platz, 2013; Khan and Boyd, 1969a; Manninen *et al.*, 2006) (Table 1). Alleles of *Rpt5* include *Rpt5.f, Spt1.r* (previously *rpt5.*k) and *Spt1.k* (previously *rpt5.r*) from CI5791/CI9819, Kombar and Rika, respectively (Abu Qamar *et al.*, 2008; Koladia *et al.*, 2017a; Manninen *et al.*, 2000, 2006; Richards *et al.*, 2016) (Table 1). Additional loci that may also be part of the *Rpt5* locus and therefore contain additional alleles include *QRpts6L*, *QRpt6*, *AL_QRptt6‐1, Rpt‐Nomini* and *Rpt‐CIho2291* in Halcyon (Raman *et al.*, 2003), TR251 (Grewal *et al.*, 2012), Lavrans (Wonneberger *et al.*, 2017a), Nomini and CIho2291 (O’Boyle *et al.*, 2014), respectively (Table 1), as well as undesignated QTLs from Steptoe (Steffenson *et al.*, 1996), HOR 9088 (Richter *et al.*, 1998), Kaputar, ND11213 (Cakir *et al.*, 2003; Emebiri *et al.*, 2005; Liu *et al.*, 2015), Chevron (Ma *et al.*, 2004), SM89010 (Friesen *et al.*, 2006), M129 (St. Pierre *et al.*, 2010), Baudin (Cakir *et al.*, 2011), WPG8412, Pompadour, Stirling (Gupta *et al.*, 2011), Falcon (Islamovic *et al.*, 2017), H602 (Hisano *et al.*, 2017) and UVC8 (Martin *et al.*, 2018) (Table S1).

The *Rpt5* locus has been reported by multiple studies and is considered to be a complex locus that is highly important in the *P. teres* f. *teres–*barley interaction (Abu Qamar *et al.*, 2008; Adawy *et al.*, 2013; Adhikari *et al.*, 2019; Cakir *et al.*, 2011, 2003; Dontsova *et al.*, 2018; Emebiri *et al.*, 2005; Friesen *et al.*, 2006; Grewal *et al.*, 2012, 2008; Gupta *et al.*, 2011, 2010; Koladia *et al.*, 2017a; Liu *et al.*, 2015; Ma *et al.*, 2004; Manninen *et al.*, 2000, 2006; Martin *et al.*, 2018; Molnar *et al.*, 2000; Novakazi *et al.*, 2019; O’Boyle *et al.*, 2014; Raman *et al.*, 2003; Rau *et al.*, 2015; Richards *et al.*, 2016; Richter *et al.*, 1998; Rozanova *et al.*, 2019; Spaner *et al.*, 1998; St. Pierre *et al.*, 2010; Steffenson *et al.*, 1996; Wonneberger *et al.*, 2017b, 2017a). Abu Qamar *et al. *(2008) found that Rika was resistant to *P. teres* f. *teres* isolate 15A and susceptible to isolate 6A, whereas for Kombar the reciprocal reactions occurred in that Kombar was resistant to 15A and susceptible to 6A. The two alleles *rpt5.k.* and *rpt5.r* were originally designated as recessive resistance due to a 1:3 resistant:susceptible segregation ratio and therefore the requirement of a homozygous state of alleles for resistance (Abu Qamar *et al.*, 2008). The *Rpt5* region that conferred susceptibility was delimited to a *c.* 5.9 cM region in a 118‐progeny doubled haploid (DH) population derived from Rika × Kombar (Abu Qamar *et al.*, 2008). While attempting to map the susceptibility loci in Rika/Kombar, a single Rika × Kombar progeny line was resistant to both 15A and 6A, and a single progeny line was susceptible to both 15A and 6A. These putative recombinants led to the hypothesis that two closely linked genes, *rpt.r* and *rpt.k* were held in repulsion *c.* 1.8 cM apart and were responsible for susceptibility (Abu Qamar *et al.*, 2008). Under a two‐gene scenario, *rpt.r* and *rpt.k* would each be responsible for initiating a susceptible response to an effector present in each parental isolate (Abu Qamar *et al.*, 2008). Using the same Rika × Kombar DH population (Abu Qamar *et al.*, 2008), 15 expressed sequence tag‐derived markers were added to the genetic map to refine the chromosome 6H region to a *c.* 3.3 cM genetic interval and the two suspected genes to a *c.* 1.6 cM genetic interval (Liu *et al.*, 2010). Liu *et al. *(2010) concluded that the *rpt.r/rpt.k* region was most likely located close to the centromere on the long arm of chromosome 6H with high synteny to chromosomes 2 and 3 of rice and *Brachypodium*, respectively. The annotation list of the putative *rpt5* region contained two leucine‐rich repeat (LRR) receptor‐like kinases, which are often associated with plant disease resistance (Liu *et al.*, 2010).

In addition, the *Rpt5* locus has also been designated *Susceptibility* *to* *P. teres 1* (*Spt1*), indicating the dominant nature of susceptibility to *P. teres* f. *teres* in some barley lines (Richards *et al.*, 2016) (Table 1). The standard nomenclature indicated *Reaction to P. teres* (*Rpt*), with capital *R* indicating dominant resistance and lower case *r* indicating recessive resistance (Franckowiak and Platz, 2013), and did not allow for the designation of dominant susceptibility. Therefore, both designations have been used for the *Rpt5/Spt1* locus since the locus may provide dominant resistance in the case of CI5791 or dominant susceptibility in Rika/Kombar depending on the allele–effector combination (Abu Qamar *et al.*, 2008; Manninen *et al.*, 2006; Richards *et al.*, 2016; Shjerve *et al.*, 2014). Using immortal critical recombinants (ICRs) of the previously defined *Rpt5/Spt1* locus, the region was fine mapped to a *c.* 0.24 cM interval, equating to a *c.* 466 kb genomic region of *Brachypodium* with 62 annotated genes (Richards *et al.*, 2016). The genomic region of *Brachypodium* translated to *c.* 9.5 Mb of physical sequence in the barley genome containing 39 high‐confidence genes, leading to the hypothesis that the locus contained a single, two tightly linked or an ‘island’ of susceptibility genes that interact with effectors to varying specificities depending on the model (Richards *et al.*, 2016). A total of six of the genes within the *Rpt5/Spt1* locus coded for immunity receptor‐like proteins that could be responsible for a susceptible response (Richards *et al.*, 2016). However, allelic analysis of candidate genes suggested that a divergent allelic series of a single gene rather than the two tightly linked genes or a ‘susceptibility island’ could be responsible for different effector specificities for the complex interactions with *P. teres* (Richards *et al.*, 2016). In addition, the putative Rika × Kombar recombinants that were either resistant or susceptible to both 15A and 6A were later found to be suspect; therefore, this result no longer ruled out the hypothesis of one allelic gene at the *Rpt5/Spt1* locus as concluded by Richards *et al. *(2016).

A separate approach lending support that *Rpt5/Spt1* encoded a disease resistance gene that was targeted by a proteinaceous necrotrophic effector was shown by Liu *et al. *(2015) where the protein designated PttNE1 interacted with a gene at the same 6H (*Rpt5/Spt1*) locus and designated *SPN1*. In this study, the PttNE1–*SPN1* interaction accounted for 31% of the disease variation (Liu *et al.*, 2015). The isolates 15A, 0‐1, LDN07 Pt‐5, ND89‐19 and NB022 all exhibited a compatible reaction mapping to the *SPN1* locus (Liu *et al.*, 2015). In addition, the major QTL responsible for the isolate JPT9901 did not co‐localize with *SPN1* but distally at a distinct locus, suggesting additional genes are harboured on chromosome 6H (Liu *et al.*, 2015). This single *Rpt5/Spt1* locus exhibits interactions that are reminiscent of the gene‐for‐gene model (Flor, 1971, 1955) and inverse gene‐for‐gene model (Friesen *et al.*, 2007) in parallel. Therefore, the interaction between barley and *P. teres* can follow typical biotrophic models such as the gene‐for‐gene model (Flor, 1971, 1955) resulting in effector‐triggered immunity, as well as an inverse gene‐for‐gene model (Friesen *et al.*, 2007) where necrotrophic effectors lead to necrotrophic effector‐triggered susceptibility (Liu *et al.*, 2015; Richards *et al.*, 2016).

The *Rpt7* locus confers resistance to *P. teres* f. *teres,* located on the long arm of chromosome 4H in Halcyon (Raman *et al.*, 2003; Read *et al.*, 2003) (Table 1). Currently, it is unknown whether additional QTLs reported on chromosome 4H in Steptoe (Steffenson *et al.*, 1996), Sloop (Lehmensiek *et al.*, 2007), TR251 (Grewal *et al.*, 2008), OUH602 (Yun *et al.*, 2005), Arena/HOR9088 (Richter *et al.*, 1998), PostxViresa/HOR9484 (König *et al.*, 2014, 2013), Harrington/TR306 (Spaner *et al.*, 1998) and Falcon (Islamovic *et al.*, 2017) are at the same locus (Table S1). However, a second locus designated *Rpt7* was identified by Tamang *et al. *(2015) conferring seedling resistance to two isolates of *P. teres* f. *maculata*, which will be covered later*.* As the *Rpt7* locus on chromosome 4H was designated first, we have decided to keep to current nomenclature and the *Rpt7* locus reported by Tamang *et al. *(2015) on 7H will become undesignated (Fig. 1).

**Figure 2:**
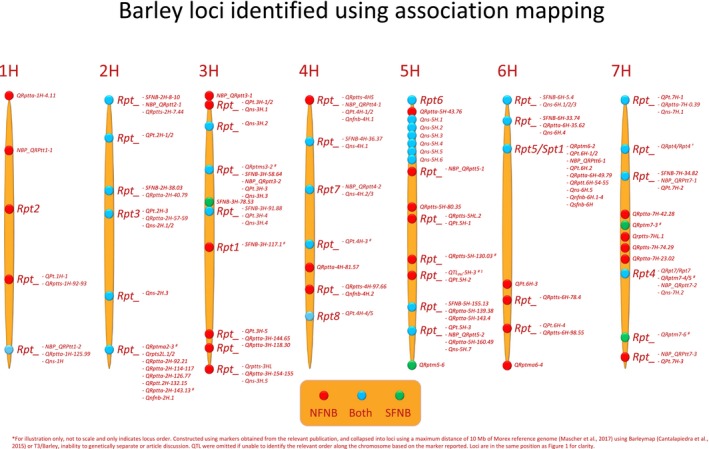
Consensus map of loci that are associated with net form net blotch (NFNB, red), spot form net blotch (SFNB, green) or both (blue) resistance/susceptibility across the barley genome identified using association mapping. Barley chromosome numbers are indicated at the top of each chromosome in sequential order. Designated loci are indicated by sequential numbering whereas previously undesignated loci are marked by an underscore (see also Table S3).

A total of seven conventional genome‐wide association studies (GWAS) have been performed to investigate *P. teres* f. *teres* resistance in barley (Adhikari *et al.*, 2019; Amezrou *et al.*, 2018; Daba *et al.*, 2019; Novakazi *et al.*, 2019; Richards *et al.*, 2017; Rozanova *et al.*, 2019; Wonneberger *et al.*, 2017b). Investigation of resistance to a set of diverse global isolates was performed on the Barley Core Collection (Muñoz‐Amatriaín *et al.*, 2014; Richards *et al.*, 2017), Nordic Barley Panel (Wonneberger *et al.*, 2017b), ICARDA AM‐2014 Panel (Amezrou *et al.*, 2018), Ethiopian, ICARDA and NDSU Barley Panel (Daba *et al.*, 2019), Siberian Barley Panel (Rozanova *et al.*, 2019), Ethiopian and Eritrean Barley Collection (Adhikari *et al.*, 2019) and Vavilov Research Institute Collection (Novakazi *et al.*, 2019). Between 7 and 31 unique genomic loci were identified in each GWAS (Fig. 2 and Table S1). Richards *et al. *(2017) revealed that the majority of the markers significantly associated with NFNB resistance localized to the centromeric region of chromosome 6H, further indicating the importance of this region.

Vatter *et al. *(2017) performed a nested association mapping (NAM) study of barley resistance to *P. teres* f. *teres*. Vatter *et al. *(2017) used reaction type (Tekauz, 1985) and average ordinate to measure the infection of *P. teres* f. *teres* on the Halle Exotic Barley 25 (HEB‐25) population (Maurer *et al.*, 2015), identifying 24 QTLs (Fig. 2). In both reaction type and average ordinate phenotyping, a QTL on chromosome 2H was responsible for the most phenotypic variation at 9.23% and 14.88%, respectively (Vatter *et al.*, 2017), but this was not the same as the *Rpt3* locus. The *Rpt5/Spt1* locus was identified in the NAM approach but only contributed 0.08% and 0.39% of the phenotypic variation for reaction type and average ordinate phenotyping, respectively (Vatter *et al.*, 2017). The low level of phenotypic variation explained by the *Rpt5/Spt1* locus could be because few of the founding barley lines used in the HEB‐25 population contained alleles that provided resistance to *P. teres* f. *teres*.

The most novel approach to mapping resistance QTLs in barley to *P. teres* f. *teres* used exome QTL‐seq (Hisano *et al.*, 2017). For exome QTL‐seq, 10 highly resistant and 10 susceptible lines were pooled separately prior to exome capture library construction and sequencing (Hisano *et al.*, 2017). Plotting the single nucleotide polymorphisms (SNPs) for QTL mapping identified two QTLs on chromosome 3H and one on chromosome 6H from barley line H602 (Hisano *et al.*, 2017). Hisano *et al. *(2017) found that there were twice as many SNPs on chromosomes 3H and 6H within the resistant bulk sample compared to the number within the susceptible bulk sample, suggesting that these loci were undergoing diversifying selection resulting in resistant lines, potentially to avoid recognition of *P. teres* effectors that result in programmed cell death. The results of Hisano *et al. *(2017) are similar to those of Gupta *et al. *(2010), Koladia *et al. *(2017a) and Liu *et al. *(2015). The sheer number of reports of QTLs located at the centromere of chromosome 6H, now referred to as *Rpt5/Spt1*, warrants validation and characterization. Research to determine the alleles and validation of *Rpt5/Spt1* in an array of barley lines is currently underway (Brueggeman *et al*., unpublished).

### Genetics of barley reactions to *P. teres* f. *maculata*

Originally, the genetics conferring resistance to *P. teres* f. *maculata* contained a total of three major designated loci and therefore had been considered less complex in comparison to the *P. teres* f. *teres–*barley interaction. The continuous distribution of *P. teres* f. *maculata* infection responses indicate that quantitative loci in barley play a part in *P. teres* f. *maculata* resistance (Burlakoti *et al.*, 2017) and occasionally previously identified resistance loci to *P. teres* f. *teres* have been found effective against *P. teres* f. *maculata*, although it is unconfirmed if the same genes are conferring resistance/susceptibility. All three of the major barley loci conferring resistance to *P. teres* f. *maculata* confer seedling resistance (Liu *et al.*, 2011) and are designated *Rpt4, Rpt6* and *Rpt8,* respectively (Table 1). The *Rpt4* locus located on chromosome 7H was found in Galleon, CI9214, Keel, Tilga, Chebec (Williams *et al.*, 2003, 1999), PI67381, PI84314 (Tamang *et al.*, 2019) and possibly TR251 (Grewal *et al.*, 2008). The *Rpt6* locus was found on the short arm of chromosome 5H in CI9819 (Burlakoti *et al.*, 2017; Manninen *et al.*, 2006) and *Rpt8* was found on chromosome 4H in Q21861 (Franckowiak and Platz, 2013; Friesen *et al.*, 2006).

Breeding lines in Australia that used *Rpt4* resistance were found to have lower levels of resistance to *P. teres* f. *maculata* than expected, indicating that additional genes were required for adult plant resistance (APR) (Williams *et al.*, 2003). Although *Rpt4* was found to contribute to APR in a Galleon × Huruna Nijo population, *Rpt4* had a much smaller effect in the adult stage when compared to *Rpt4* seedling resistance (Williams *et al.*, 2003). At least three APR QTLs were mapped, one QTL on each of chromosomes 4H and 5H, and one to three QTLs on chromosome 7H (Williams *et al.*, 2003) (Table S2). The barley lines Galleon, VB9104, CI9214, Kell and Chebec were found to have an APR locus on chromosome 7H, whereas Galleon and VB9104 also contained the APR loci on chromosomes 4H and 5H (Williams *et al.*, 2003). The one to three APR loci on chromosome 7H mapped distal to *Rpt4* (Williams *et al.*, 2003), but based on the interval mapping approach used, all three loci are closely linked or overlap so it is difficult to conclude whether there are one, two or three APR loci in addition to *Rpt4*.

Four studies have used GWAS to investigate barley resistance to *P. teres* f. *maculata* (Burlakoti *et al.*, 2017; Daba *et al.*, 2019; Tamang *et al.*, 2015; Wang *et al.*, 2015)*.* Evaluations of resistance to diverse global (Tamang *et al.*, 2015), Australian (Wang *et al.*, 2015) and single American isolates (Burlakoti *et al.*, 2017; Daba *et al.*, 2019) were performed on the Barley Core Collection (Muñoz‐Amatriaín *et al.*, 2014; Neupane *et al.*, 2015; Tamang *et al.*, 2015), Northern Region Barley Breeding Program of Australia (Wang *et al.*, 2015), Upper Midwest Breeding Programs (Burlakoti *et al.*, 2017) and Ethiopian, ICARDA and NDSU Barley Panel (Daba *et al.*, 2019), respectively. A total of 27 (Tamang *et al.*, 2015), 29 (Wang *et al.*, 2015), 11 (Burlakoti *et al.*, 2017) and 1 (Daba *et al.*, 2019) unique genomic loci were identified in each GWAS, respectively (Fig. 2 and Table S2).

The previously identified loci included *QRpts4*, *QRpt6* corresponding to *Rpt5/Spt1* (Grewal *et al.*, 2008), *Rpt4*, *Rpt6*, *Rpt7* (Williams *et al.*, 2003) and QTLs on 4H (Friesen *et al.*, 2006) corresponding to *Rpt8* (Tamang *et al.*, 2015)*.* The *Rpt7* locus reported by Tamang *et al. *(2015) on 7H was postulated to be the undesignated APR locus discovered by Williams *et al. *(2003), and therefore should remain an undesignated locus as the *Rpt7* locus was previously designated on chromosome 4H (Table 1). In addition, the *QRpt7* locus was previously reported by Grewal *et al. *(2008) and was established to be *Rpt4* on chromosome 7H*. QRpts4* (Grewal *et al.*, 2008) and APR on chromosome 4H (Williams *et al.*, 2003) may be the same locus as *Rpt7* on chromosome 4H. The APR QTL located on chromosome 5H (Williams *et al.*, 2003) is not *Rpt6* and remains undesignated.

Four highly significant QTLs (*QRptm7‐4, QRptm7‐6, QRptm7‐7* and *QRptm7‐*8) map to a 36 cM region that approximately encompasses the *Rpt4* locus on chromosome 7H (Wang *et al.*, 2015), similar to results reported by Williams *et al. *(2003). However, it is unknown if QTLs on chromosome 7H are multiple linked QTLs, multiple alleles of a single QTL or an artefact of population structure (Wang *et al.*, 2015). Analysis of linkage decay of the QTLs located on chromosome 7H suggests that the four significant QTLs are independent of each other, but further verification is required (Wang *et al.*, 2015).

Burlakoti *et al. *(2017) studied the effect of two‐ and six‐row barley, concluding that the proportion of six‐row barley (43%) resistant to *P. teres* f. *maculata* was higher than that of the two‐row barley (13%) tested. This difference is in contrast to Wang *et al. *(2015), who had comparable numbers of resistant lines (40–65%) in Australian two‐ and six‐row barley. The most significant QTL identified was novel, mapped to chromosome 2H and accounted for 4.3–6.6% of the phenotypic variation (Burlakoti *et al.*, 2017). Daba *et al. *(2019) identified a single novel QTL on chromosome 6H, but this is attributed to most lines being susceptible to *P. teres* f. *maculata.* The association mapping studies of Tamang *et al. *(2015), Wang *et al. *(2015) and Burlakoti *et al. *(2017) indicated that the genetic interaction of *P. teres* f. *maculata* and barley was more complex than originally hypothesized. In addition, novel QTLs and therefore sources of resistance can be identified with the power of association mapping to evaluate more accessions in parallel.

Recently, studies to identify new sources of resistance to *P. teres* f. *maculata* have been undertaken by Çelik Oğuz *et al. *(2017) and Gyawali *et al. *(2018). Çelik Oğuz *et al. *(2017) found two six‐row landraces in Turkey that were resistant to all (three *P. teres* f. *teres* and three *P. teres* f. *maculata*) isolates tested that could be used in breeding programmes. Gyawali *et al. *(2018) screened 340 diverse ICARDA barley lines using one *P. teres* f. *maculata* isolate to identify 12 lines that were highly resistant and stable across environments to be used for resistance breeding. However, without mapping and subsequent allelic analysis it is unknown what resistance loci or alleles are present.

### Effectors of *P. teres*

Effectors are molecules that interact with the host to manipulate the host response, and encompass secondary metabolites, peptides, small secreted proteins, enzymes and more recently small RNAs (Toruño *et al.*, 2016). Effectors can be identified in multiple ways, including forward and reverse genetic approaches, including traditional genetic mapping, genomic mining and proteomics/metabolomic approaches. A genetic mapping approach to effector discovery is labour‐intensive and includes population development, genotyping and phenotyping but can generate highly useful candidate gene lists to functionally validate effectors that segregate within a given population. The use of genetic mapping is less effective in finding highly conserved effectors that do not segregate between parents of a population or those that become fixed within a natural population. Genomic mining or proteomic/metabolomic approaches can be used to identify these highly conserved effectors but result in large candidate lists of genes that need to be functionally validated.

For the successful colonization by necrotrophic fungi such as *P. teres* f. *teres* and *P. teres* f. *maculata*, necrosis and chlorosis of plant tissue is associated with a compatible reaction (reviewed in Liu *et al.*, 2011). Chlorosis surrounding the necrotic lesion is often associated with diffusible phytotoxic secondary metabolites (Keon and Hargreaves, 1983; Sarpeleh *et al.*, 2007). Four toxins, *N*‐(2‐amino‐2‐carboxyethyl)‐aspartic acid, anhydroaspergillomarasmine A, aspergillomarasmine A and aspergillomarasmine B, were isolated and their pathways subsequently identified (Bach *et al.*, 1979; Friis *et al.*, 1991; Sarpeleh *et al.*, 2009; Smedegård‐Petersen, 1977) and used to screen for resistance to *P. teres* (Sharma, 1984; Weiergang *et al.*, 2002). All four toxins identified were of the marasmine class that chelate iron ions and have been shown to be light‐ and temperature‐dependent, indicating a potential role targeting organelles such as the chloroplast (Sarpeleh *et al.*, 2009). Secondary metabolites do not appear to be isolate‐specific and little host specificity exists apart from lines being either sensitive or insensitive (Sarpeleh *et al.*, 2009, 2007).

Multiple studies have shown that different virulence profiles exist within *P. teres* populations around the globe (Akhavan *et al.*, 2016; Arabi *et al.*, 2003; Cromey and Parkes, 2003; Fowler *et al.*, 2017; Gupta and Loughman, 2001; Harrabi and Kamel, 1990; Jalli and Robinson, 2000; Jonsson *et al.*, 1997; Khan and Boyd, 1969b; Liu *et al.*, 2012; McLean *et al.*, 2010b; Robinson and Jalli, 1996; Sato and Takeda, 1997; Steffenson and Webster, 1992; Tekauz, 1990; Wu *et al.*, 2003). Virulence variation among isolates on different barley lines appears to originate from their segregating effector repertoire, many of which are now believed to be proteinaceous in nature (Liu *et al.*, 2015; Richards *et al.*, 2016). Virulent *P. teres* f. *teres* and *P. teres* f. *maculata* isolates have increased production of proteases, possibly for faster establishment of the pathogen and increased sugar acquisition compared to less virulent isolates (Dikilitas *et al.*, 2018). Two papers from the same group proposed that variations in virulence could be explained by fungal growth, ability to deliver effectors and differentially expressing effectors (Ismail *et al.*, 2014a, 2014b). Ismail *et al. *(2014a) used six *P. teres* f. *teres* isolates and found that the four more virulent isolates had greater conidial germination and appressoria formation compared to the less virulent isolates yet extracted proteinaceous effectors from all isolates were able to induce necrosis. Three proteinaceous effectors were postulated in the virulent *P. teres* f. *teres* isolate 32/98 as PttXyn11A, PttCHFP1 and PttSP1 that showed homology to proteins involved in plant disease interactions (Ismail *et al.*, 2014b). Using a combination of one‐ and two‐dimensional electrophoresis, Ismail and Able (2016) identified 63 proteins that were produced by 28 virulent isolates. Ismail and Able (2017) subsequently identified a transition between colonization and necrotrophy at approximately 48 h based on the *in planta* gene expression analysis of 222 proteins. As no functional validation was reported on these candidate genes/proteins, it is not yet known whether any of these are biologically relevant to the *P. teres* f. *teres–*barley interaction.

**Figure 3:**
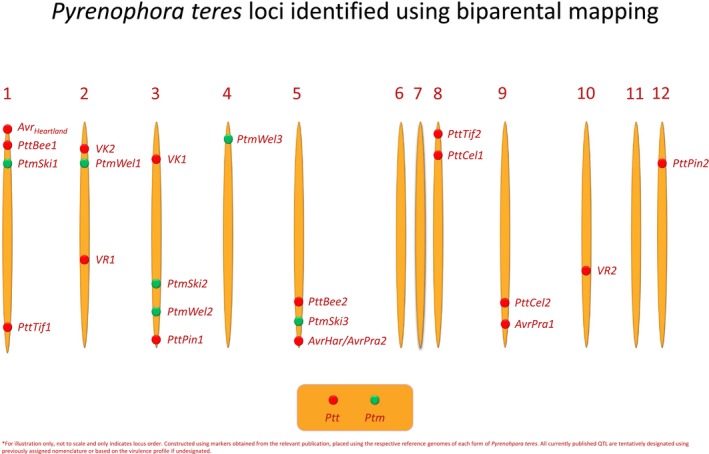
Map of *Pyrenophora teres* f. *teres* (Ptt, red) and *P. teres* f. *maculata* (Ptm, green) effectors across the *P. teres* genome identified using biparental mapping. Chromosome numbers are indicated at the top of each chromosome.

A proteinaceous necrotrophic effector, PttNE1, was isolated from intercellular wash fluid by Liu *et al. *(2015) using the susceptible barley line Hector inoculated with *P. teres* f. *teres* isolate 0‐1. PttNE1 exhibited direct association with disease and interacted with *SPN1*, and therefore Liu *et al. *(2015) concluded that host sensitivity resulted from necrotrophic effector‐triggered susceptibility, but the pathogen gene *PttNE1* was not mapped in *P. teres* f. *teres*. The first mapped effector implicated in the *P. teres* f. *teres–*barley system, designated *AvrHar*, was identified by Weiland *et al. *(1999) in the *P. teres* f. *teres* 15A × 0‐1 population. The 15A allele of *AvrHar* was proposed to contribute low virulence on the barley line Harbin (Weiland *et al.*, 1999) (Table 2). In an expanded population of 15A × 0‐1, the 15A allele of the *AvrHar* locus also conferred avirulence on Tifang and Canadian Lake Shore (Lai *et al.*, 2007). In addition, two QTLs, designated *AvrPra1* and *AvrPra2,* from 0‐1 were mapped and found to be functionally redundant to confer avirulence on Prato (Lai *et al.*, 2007) (Table 2). The *AvrHar* and *AvrPra2* loci mapped to the same locus on linkage group 7 (chromosome 5) (Lai *et al.*, 2007; Wyatt *et al*., 2018), but whether the locus contains two tightly linked genes or alleles of the same gene is yet to be confirmed (Fig. 3). The *AvrPra1* locus was mapped to linkage group 1 (Lai *et al.*, 2007), currently designated chromosome 9 based on the 0‐1 assembly (Fig. 3, Wyatt *et al*., 2018). A separate avirulence gene, designated *Avr_Heartland_*, was identified in a 67‐progeny mapping population with the Canadian avirulent isolate WRS 1906 and the virulent isolate WRS 1607 (Beattie *et al.*, 2007) (Table 2).

**Table 2:** Current *Pyrenophora teres* f. *teres* and *P. teres* f. *maculata* genes defined in the *P. teres–*barley pathosystem including the current and previous synonyms, known alleles, and which barley lines the allele is virulent or avirulent on along with phenotypic variation, the *P. teres* chromosome location of the effector and barley chromosome target

Locus	Previous synonym	Alleles*	Virulent on†	Avirulent on‡	*R* ^2^ (%)§	Location‖	Target
*AvrHar*		*AvrHar* (15A)*, AvrPra2* (0‐1)	Harbin, Tifang, Canadian Lake Shore^b^, Prato^a^	Harbin, Tifang, Canadian Lake Shore^a^, Prato^b^	–	Chr 5	–
*AvrPra1*		*AvrPra1* (0‐1)	Tifang, Canadian Lake Shore	Prato	–	Chr 9	–
*Avr_Heartland_*		*Avr_Heartland_* (WRS 1906)	Harrington	Heartland	–	Chr 1	–
*VK1*		*VK1* (15A)	Kombar	Rika	0.26	Chr 3	*Rpt5/Spt1*
*VK2*		*VK2* (15A)	Kombar	Rika	0.19	Chr 2	*Rpt5/Spt1*
*VR1*		*VR1* (6A)	Rika	Kombar	0.35	Chr 2	*Rpt5/Spt1*
*VR2*		*VR2* (6A)	Rika	Kombar	0.20	Chr 10	*Rpt5/Spt1*
*PttTif1*		*PttTif1* (FGOH04Ptt‐21)	Tifang, Manchurian, CI 4822	Beecher	0.45–0.67	Chr 1	–
*PttTif2*		*PttTif3* (FGOH04Ptt‐21)	Tifang	Manchurian, CI 4822, Beecher, Pinnacle, Celebration, Hector, Stellar	0.026	Chr 8	–
*PttBee1*		*PttBee1* (FGOH04Ptt‐21)	Beecher	Tifang, Manchurian, CI 4822, Pinnacle, Celebration, Hector, Stellar	0.56	Chr 1	–
*PttBee2*		*PttBee2* (FGOH04Ptt‐21)	Beecher	Tifang, Manchurian, CI 4822, Pinnacle, Celebration, Hector, Stellar	0.17	Chr 5	–
*PttPin1*		*PttPin1* (BB25)	Pinnacle	Tifang, Manchurian, CI 4822, Beecher, Celebration, Hector, Stellar	0.49	Chr 3	–
*PttPin2*		*PttPin2* (FGOH04Ptt‐21)	Pinnacle	Tifang, Manchurian, CI 4822, Beecher, Celebration, Hector, Stellar	0.11	Chr 12	–
*PttCel1*		*PttCel1* (FGOH04Ptt‐21)	Celebration, Tifang	Manchurian, CI 4822, Beecher, Pinnacle, Hector, Stellar	0.066–0.17	Chr 8	–
*PttCel2*		*PttCel2* (FGOH04Ptt‐21)	Celebration	Tifang, Manchurian, CI 4822, Beecher, Pinnacle, Celebration, Hector, Stellar	0.17	Chr 9	–
*PttHec1*		*PttHec1* (FGOH04Ptt‐21)	Hector, Stellar	Tifang, Manchurian, CI 4822, Beecher, Pinnacle, Celebration	0.11–0.18	Chr 8	–
*PtmSki1*	*vQTL1A* *vQTL1B* *vQTL1C*	*PtmSki1* (FGOB10Ptm‐1)	Skiff, TR326 81‐82/033 Welgevallen65‐31‐36	– – –	0.21–0.23 0.21 0.34	Chr 1 Chr 1 Chr 1	– – –
*PtmSki2*	*vQTL2*	*PtmSki2* (FGOB10Ptm‐1)	Skiff	Welgevallen65‐31‐36, 81‐82/033, TR326	0.22	Chr 3	–
*PtmSki3*	*vQTL3*	*PtmSki3* (FGOB10Ptm‐1)	Skiff	Welgevallen65‐31‐36, 81‐82/033, TR326	0.20	Chr 5	–
*PtmWel1*	*vQTL4*	*PtmWel1* (SG1)	Welgevallen65‐31‐36, 81‐82/033	Skiff, TR326	0.30–0.37	Chr 2	–
*PtmWel2*	*vQTL5*	*PtmWel2* (FGOB10Ptm‐1)	Welgevallen65‐31‐36, 81‐82/033, TR326	Skiff	0.26–0.34	Chr 3	–
*PtmWel3*	*vQTL6*	*PtmWel3* (FGOB10Ptm‐1)	Welgevallen65‐31‐36	Skiff, 81‐82/033, TR326	0.20	Chr 4	–

* Isolate contributing virulent allele.

^†^ Barley line the virulent isolate is known to be virulent on, additional lines may exist, ^a^ allele 1, ^b^ allele 2.

^‡^ Barley line the virulent isolate is known to be avirulent on, additional lines may exist, ^a^ allele 1, ^b^ allele 2.

^§^ QTL effects containing ranges mean that the experiments were performed for multiple locations or multiple isolates and the effects for individual treatments fall into this range.

‖ Inferred through current genome assembly and chromosome numbering (Wyatt *et al*., 2018).

— No information is available for this entry.

Afanasenko *et al. *(2007) proposed the presence of a suppressor of avirulence genes where virulence arose in 30 out of 84 progeny from an avirulent by virulent cross on the barley line Diamond. Based on an avirulent: virulent segregation ratio of 9:7, Afanasenko *et al. *(2007) suggested the presence of two avirulence genes and one suppressor gene. Based on phenotypic ratios alone, Afanasenko *et al. *(2007) proposed that up to four avirulence genes and up to two suppressor genes in various combinations were present in four *P. teres* f. *teres* crosses, but the corresponding loci were not mapped.

Shjerve *et al. *(2014) was the first study to begin using a restriction‐site associated‐digest–genotyping‐by‐sequencing (RAD‐GBS) approach detailed by Leboldus *et al. *(2015) that would allow the rapid construction of genetic maps by subsequent studies including Koladia *et al. *(2017b) and Carlsen *et al. *(2017) in *P. teres* f. *teres* and *P. teres* f. *maculata*, respectively. The RAD‐GBS approach facilitated the mapping of four QTL regions that have now been assigned chromosomal designations based on the recently updated 0‐1 reference genome assembly (Wyatt *et al*., 2018) including *VK1* and *VK2* (*Virulence* *on* *Kombar 1* and *2*) and *VR1* and *VR2* (*Virulence* *on* *Rika 1* and *2*). *VK1* localized to chromosome 3, *VK2* and *VR1* to separate loci on chromosome 2 and *VR2* on chromosome 10 (Fig. 3). Isolate 15A harboured the virulence alleles at *VK1* and *VK2* with each conferring virulence on Kombar but lacking virulence on Rika (Shjerve *et al.*, 2014) (Table 2). The isolate 6A harboured virulence alleles at *VR1* and *VR2* and the reciprocal occurred, where *VR1* and *VR2* confer virulence on Rika but lacked virulence on Kombar (Shjerve *et al.*, 2014) (Table 2). In addition, Shjerve *et al. *(2014) further characterized the progeny isolates of the 15A × 6A population to discover that *VK1*, *VK2* and *VR2* all targeted the same *Spt1* host region on chromosome 6H. Isolates containing *VK1* or *VK2* alone were each virulent on Kombar containing *Spt1.k*, whereas the isolate that contained *VR2* alone was avirulent (Shjerve *et al.*, 2014)*.* Conversely the isolate that contained *VR2* alone was virulent on Rika that contained *Spt1.r* but isolates that contained *VK1* and *VK2* alone were avirulent (Shjerve *et al.*, 2014).

Koladia *et al. *(2017b) identified nine unique QTLs in the *P. teres* f. *teres* cross between the Danish isolate BB25 and the North Dakotan isolate FGOH04Ptt‐21, of which only one of the virulence alleles was conferred by BB25 and eight were conferred by FGOH04Ptt‐21 (Table 2). A major QTL (*PttTif1*) with the virulent allele from FGOH04Ptt‐21 found on linkage group 10.1 (chromosome 1) contributed as much as 74% of the phenotypic variation on three lines (Manchurian, Tifang and CI4822) (Koladia *et al.*, 2017b) (Fig. 3). The second major locus (*PttBee1*) conferring virulence from FGOH04Ptt‐21 was found on linkage group 1.1 (chromosome 1), contributing 56% of the phenotypic variation on the barley line Beecher (Koladia *et al.*, 2017b) (Fig. 3). The BB25 isolate was only virulent on barley line Pinnacle with the BB25 virulence allele (*PttPin1*) located on linkage group 5.1 (chromosome 3), which explained 49% of the phenotypic variation (Koladia *et al.*, 2017b) (Fig. 3). The virulence alleles of the remaining six loci all contributed less than 18% of the phenotypic variation and were distributed across multiple chromosomes (Koladia *et al.*, 2017b). Currently, effector candidate genes underlying these major QTLs are being evaluated.

Carlsen *et al. *(2017) is currently the only study to map *P. teres* f. *maculata* virulence. The FGOB10Ptm‐1 × SG1 population was inoculated on commonly used SFNB differential barley lines Skiff, TR326, 81‐82/033 and PI392501 with a total of six loci identified across five of the 12 linkage groups (Carlsen *et al.*, 2017) (Fig. 3 and Table 2). FGOB10Ptm‐1 and SG1 contributed the virulence allele at five and one QTL, respectively (Carlsen *et al.*, 2017). The QTL with the virulence allele contributed by SG1 (vQTL4, *PtmWel1*) on linkage group 1.1 (chromosome 2) accounted for 30–37% of the phenotypic variation on barley lines 81‐82/033 and PI392501, whereas the largest effect QTL with the virulence allele contributed by FGOB10Ptm‐1 (vQTL5, *PtmWel2*) on linkage group 5.1 (chromosome 3) accounted for 26–34% of the phenotypic variation on barley lines 81‐82/033, TR326 and PI392501 (Carlsen *et al.*, 2017) (Fig. 3 and Table 2). We have assigned tentative designations for this review until a robust naming nomenclature is established.

### Advancement of genomic resources in barley and *P. teres*

The first assembled genome of barley was published by IBGSC (2012) using the American six‐row malting cultivar Morex. Again, Morex was used for a high‐quality reference assembly using chromosome conformation capture (Beier *et al.*, 2017; Mascher *et al.*, 2017), with subsequent analysis in the repetitive elements (Wicker *et al.*, 2017). Additional lines that have been sequenced include the American two‐row spring feed barley line Bowman, the German two‐row spring malting barley line Barke, the Scottish two‐row winter malting barley line Igri (IBGSC, 2012), the Japanese two‐row spring malting barley line Haruna Nijo (IBGSC, 2012; Sato *et al.*, 2016), and the Tibetan hulless cultivars Lasa Goumang (Zeng *et al*., 2015) and Zangqing320 (Dai *et al.*, 2018; Nyima *et al.*, 2018), but no published studies have compared these released barley genomes (Monat *et al.*, 2018). Multiple studies have revealed genomic signatures of domestication using exome sequencing of 267 barley accessions (Russell *et al.*, 2016), targeted resequencing of 433 barley accessions (Pankin *et al.*, 2018) and whole‐genome sequencing of 172 barley accessions (Zeng *et al.*, 2018). In the future we will be able to generate pan‐genomic resources that consolidate resistance loci to *P. teres* from different studies into their respective allelic series. Currently, large‐scale barley genome sequencing for pan‐genomic resources is hampered by the large amount of repetitive content in the barley genome (Monat *et al.*, 2018). In this review, we consolidated mapped loci that have not currently been proven to be separate loci.

A wealth of *P. teres* genomic resources has been generated since the first genetic marker studies evaluating virulence/avirulence. The first *P. teres* genome was of the Canadian *P. teres* f. *teres* isolate 0‐1 using Illumina paired‐end reads (Ellwood *et al.*, 2010). The size of 0‐1 was initially estimated at 41.95 Mb with an average sequence coverage of 20×, confirming the presence of at least nine chromosomes based on pulsed‐field gel electrophoresis and germ tube burst visualization (Ellwood *et al.*, 2010). Wyatt *et al*. (2018) subsequently confirmed 12 chromosomes with a reference quality assembly of the same 0‐1 isolate using PacBio long‐read single‐molecule real‐time (SMRT) sequencing. The 0‐1 assembly produced a reported genome size of 46.5 Mb at an average coverage of 200× (Wyatt *et al*., 2018). Current genome assemblies include an additional eight *P. teres* f. *teres* isolates (W1‐1, NB29, NB85, NB73, 15A, 6A, FGOH04Ptt‐21 and BB25) and two *P. teres* f. *maculata* isolates (FGOB10Ptm‐1 and SG1) (Syme *et al.*, 2018; Wyatt *et al.*, 2019). Genome sizes currently are in the range of 39.27–41.28 Mb for *P. teres* f. *maculata* and 46.31–51.76 Mb in *P. teres* f. *teres*, concluding that *P. teres* f. *maculata* has a smaller genome due mostly to repetitive elements (Syme *et al.*, 2018).

Due to the diversity of the natural *P. teres* f. *teres* population, evident from the mapped effectors mentioned earlier, a pan‐genomic sequencing approach of four *P. teres* f. *teres* isolates 15A, 6A, FGOH04Ptt‐21 and BB25 was used to evaluate the genomic architecture and gene content in comparison to 0‐1 (Wyatt *et al.*, 2019). Each *P. teres* f. *teres* isolate was predicted to contain approximately 200 effectors using EffectorP (Wyatt *et al.*, 2019), but EffectorP prediction is known to underestimate effectors due to stringent criteria. The majority of published virulence QTLs have been identified in subtelomeric regions where chromosome rearrangements and repetitive elements are found at higher frequency, allowing rapid development of polymorphisms (Wyatt *et al.*, 2019). Wyatt *et al. *(2019) showed that 14 of 15 currently published *P. teres* f. *teres* QTLs span accessory genomic compartments with ten of 14 accessory genomic compartment QTLs specifically localizing to subtelomeric regions. This observation highlights the importance of the subtelomeric regions as drivers of virulence for *P. teres* f. *teres*. Additionally, a whole chromosome fusion was found between chromosomes 1 and 2 of isolate BB25, which is predicted to be a recent event and not inherited through ancestry and lost in other lineages (Wyatt *et al.*, 2019). Chromosome fusions have been recently identified in the closely related species *P. tritici‐repentis* in reference isolate M4 and may be a common feature in the *Pyrenophora* genus (Moolhuijzen *et al.*, 2018).

## Conclusion and Future Direction

Liu *et al. *(2011) stated that the following characteristics facilitate the use of *P. teres* as a candidate model organism: the worldwide economic importance of the disease, the ability to produce biparental mapping populations and clone genes from the pathogen, the amenability of the pathogen to transformation for gene validation, relatively abundant genomic resources in both the host and pathogen, and the large numbers of host mapping populations to decipher the interactions and diversity of both the host and pathogen. These points were in their infancy and have been built on since Liu *et al. *(2011). As noted, *P. teres* has become an increasing problem worldwide, leading to the expansion of pathogen and host mapping studies that have identified loci important for pathogen virulence and host resistance/susceptibility using biparental and association mapping studies. In summary, over 340 and 140 QTLs have been published in relation to barley responses to *P. teres* f. *teres* and *P. teres* f. *maculata* isolates, respectively, of which eight loci are sequentially designated from *Rpt1* to *Rpt8* on chromosomes 3H, 1H, 2H, 7H, 6H, 5H, 4H and 4H, respectively (Table 1). Despite fine‐mapping of loci such as *Rpt5/Spt1* (Richards *et al.*, 2016), delimiting loci within the barley pan‐genome remains difficult due to the multitude of studies that report large overlapping genomic regions (Figs 1 and 2). Additionally, a large proportion of identified QTLs are collapsed into 39 consensus loci, resulting in a total of 47 loci (Figs 1 and 2). In contrast, a total of 22–23 loci have been published in *P. teres* that follow a tentative nomenclature of (a)virulence on a specific barley line (Table 2). An exponential growth of genomic resources for *P. teres* has occurred since the first genome assembly reported by Ellwood *et al. *(2010), with reference quality genome assemblies and analysis (Syme *et al.*, 2018; Wyatt *et al.*, 2019). Adoption of the *P. teres–*barley pathosystem as an agriculturally significant model has allowed rapid advancement in the abundance of genomic sequence, biparental and natural populations, and marker trait associations/QTLs to validate and characterize genes in both host and pathogen. Despite some overlap in functional resistance/susceptibility loci to *P. teres* f. *teres* and *P. teres* f. *maculata* in barley, each form of *P. teres* has a novel repertoire of effectors at their disposal. Therefore, breeding for resistance in barley to *P. teres* f. *teres* and *P. teres* f. *maculata* should proceed separately based on the most effective loci to the respective local *P. teres* populations.

## Supporting information


**Table S1** Studies mapping barley resistance/susceptibility genes to *Pyrenophora teres *f. *teres. *Different populations are indicated by alternating grey scale with the parent contributing the resistance allele in bold. Plant stage indicates whether the resistance is effective at the seedling or adult stage. If a single genotype isolate is used, this is indicated by the name and country of origin, whereas natural infection is indicated by specific location. Barley chromosome, phenotypic variation and designation of each locus are displayed, if available, from the corresponding reference. The inferred locus designation is reported based on markers obtained from the relevant publication and collapsed into loci using a maximum distance of 10 Mb of the Morex reference genome (Mascher *et al*., 2017) using BarleyMap (Cantalapiedra *et al*., 2015) or T3/Barley (Fig. 1).

**Table S2** Studies mapping barley resistance/susceptibility genes to *Pyrenophora teres *f. *maculata. *Different populations are indicated by alternating grey scale with the parent contributing the resistance allele in bold. Plant stage indicates whether the resistance is effective at the seedling or adult stage. If a single genotype isolate is used this is indicated by the name and country of origin, whereas natural infection is indicated by specific location. Barley chromosome, phenotypic variation and designation of each locus are displayed if available from the corresponding reference. The inferred locus designation is reported based on markers obtained from the relevant publication and collapsed into loci using a maximum distance of 10 Mb of the Morex reference genome (Mascher *et al*., 2017) using BarleyMap (Cantalapiedra *et al*., 2015) or T3/Barley (Fig. 1).

**Table S3** Genomic positions of markers reported in biparental or association mapping studies that could be anchored to the Morex barley reference genome (Mascher *et al*., 2017), including the locus designation (from publication), the corresponding marker, the assigned chromosome and chromosome position, and the reference.

## Data Availability

Data sharing is not applicable to this article as no new data were created or analysed in this study.
