# Supplementary material for: Research advances in the Pyrenophora teres–barley interaction
Source: Mol Plant Pathol. 2019 Dec 13;21(2):272–88. doi: 10.1111/mpp.12896 (PMC6988421; doi:10.1111/mpp.12896)
Supplement: Supplementary file 1 — Table S1 Studies mapping barley resistance/susceptibility genes to Pyrenophora teres f. teres. Different populations are indicated by alternating grey scale with the parent contributing the resistance allele in bold. Plant stage indicates whether the resistance is effective at the seedling or adult stage. If a single genotype isolate is used, this is indicated by the name and country of origin, whereas natural infection is indicated by specific location. Barley chromosome, phenotypic variation and designation of each locus are displayed, if available, from the corresponding reference. The inferred locus designation is reported based on markers obtained from the relevant publication and collapsed into loci using a maximum distance of 10 Mb of the Morex reference genome (Mascher et al., 2017) using BarleyMap (Cantalapiedra et al., 2015) or T3/Barley (Fig. 1). [file MPP-21-272-s001.docx]

**Supplemental Table S1.** Studies mapping barley resistance/suceptibility genes to *Pyrenophora teres* f. *teres.* Different population are indicated by alternating grey scale with parent contributing the resistance allele in bold. Plant stage indicates whether the resistance is active at the seedling or adult stage. If a single genotype isolate is used this is indicated by the name and country of origin, whereas natural infection is indicated by specific location. Barley chromosome, phenotypic variation and designation of each locus is displayed if available from the corresponding reference. The inferred locus designation is reported based on markers obtained from the relevant publication, and collapsed into loci using a maximum distance of 10 Mb of the Morex reference genome (Mascher et al., 2017) using BarleyMap (Cantalapiedra et al., 2015) or T3/Barley (Supplemental Table 3), inability to genetically separate in mapping or article discussion if possible.

| Population (type) * | Plant Stage † | Isolate (origin) ‡ | Chr. Location | R^2^ (%) § | Locus Designation | References ‖ | Inferred Locus Designation ¶ |
| --- | --- | --- | --- | --- | --- | --- | --- |
| Parkland × **CI13663** (**CI5791**) (BC_1_F_2_) | Seedling | WRS102 (Canada) | - | - | - | Metacalfe et al 1970 | *Rpt5/Spt1 ^#^* |
| Betzes Primary Trisomics × **Tifang** (F_2_) |  | ES8, ES9, ES10 (Tunisia) | 3H | - | *Rpt1a* | Bockelman et al 1977 | *Rpt1* |
| Betzes Primary Trisomics × **CI9819** (F_2_) |  |  | 3H | - | *Rpt1b* |  | *Rpt1* |
|  |  |  | 1H | - | *Rpt2c* |  | *Rpt2* |
| Betzes Primary Trisomics × **CI7584** (F_2_) |  |  | 2H | - | *Rpt3d* |  | *Rpt3* |
| Minnesotan Breeding Program (Artificial) |  | Unknown (USA) | - | - | *-* | Wilcoxson et al 1992 | *-* |
| **Léger** × CI 9831 (DH) | Seedling | WRS102 (Canada) | 2H | - | *-* | Ho et al 1996 | *Rpt3 ^#^* |
|  |  |  | - | - | *-* |  | *-* |
|  |  |  | - | - | *-* |  | *-* |
|  |  | WRS858 (Canada) | - | - | *-* |  | *-* |
| **Steptoe** × Morex (DH) | Seedling | ND89-19 (USA) | 4P | 31 | - | Steffenson et al 1996 | *Rpt7 ^#^* |
|  |  |  | 6M | 14 | *-* |  | *Rpt5/Spt1* |
|  |  |  | 6P | 10 | *-* |  | *-* |
|  | Adult | ND89-19 (USA) | 1P | 21 | *-* |  | *Rpt2 ^#^* |
|  |  |  | 3P | 20 | *-* |  | *-* |
|  |  |  | 2P | 15 |  |  | *Rpt3 ^#^* |
|  |  |  | 3M | 16 | *-* |  | *-* |
|  |  |  | 7P | 11 | *-* |  | *-* |
| **Igri** × Franka (DH) | Seedling | WRS1240 (Canada) | 3HL | 100 | *Pt,,a* | Graner et al 1996 | *Rpt1* |
| **Harrington** × TR306 (DH) | Adult | Natural Infection (Pullman, USA; Edmonton, Brandon, Ailsa Craig, Elora, Charlottetown, Canada) | 5H | - | *-* | Spaner et al 1998 | *-* |
| Harrington × **TR306** (DH) | Adult | Natural Infection (Pullman, USA; Edmonton, Brandon, Ailsa Craig, Elora, Charlottetown, Canada) | 4H | - | *-* |  | *-* |
|  |  |  | 6H | - | *-* |  | *Rpt5/Spt1 ^#^* |
|  |  |  | 7H | - | *-* |  | *-* |
| Arena × **HOR 9088** (F_2_) | Seedling | 04/6T (-) | 3HL | 19.2 | *-* | Richter et al 1998 | *Rpt1 ^#^* |
|  |  |  | 6H | 22.6 | - |  | *Rpt5/Spt1 ^#^* |
|  |  |  | 6H | 10.3 | *-* |  | *Rpt5/Spt1 ^#^* |
| Alexis × **Sloop** (DH) | Seedling | NB34 (Australia) | 2HS | 11 | *QRpts2S* | Raman et al 2003 | *QRpts2S* |
| **Alexis** × Sloop (DH) |  |  | 3HL | 17 | *QRpts3L* |  | *Rpt1* |
| **Sloop-sib** × Alexis (RIL) | Seedling | NB34 (Australia) | 2HS | 9 | *QRpts2Sa* |  | *QRpts2S* |
|  |  |  | 2HS | - | *QRpts2Sb* |  | *Rpt3* |
| Sloop-sib × **Alexis** (RIL) |  |  | 3HL | 13 | *QRpts3L* |  | *Rpt1* |
| Arapiles × **Frankin** (DH) | Seedling | NB34 (Australia) | 2HS | 13 | *QRpts2S* |  | *QRpts2S* |
|  |  |  | 2HL | 7 | *QRpts2L* |  | *QRpts2L* |
|  |  |  | 3HL | 9 | *QRpts3Lb* |  | *QRpts3Lb* |
| **Arapiles** × Frankin (DH) |  |  | 3HL | 16 | *QRpts3La* |  | *QRpts3La* |
| Sloop × **Halcyon** (DH) | Seedling | NB50 (Australia) | 4H | 64 | *QRpts4* | ^1^Read et al 2003 | *Rpt7* |
|  |  |  | 6HL | 11 | *QRpts6L* |  | *Rpt5/Spt1* |
| Tallon × **Kaputar** (DH) | Seedling | NB52B, NB54, NB81 and NB97 (Australia) | 2H | 20-29 | *-* | Cakir et al 2003 | *Rpt3 ^#^* |
|  |  |  | 3H | 24-31 | - |  | *Rpt1* |
|  |  |  | 6H | 46-83 | - |  | *Rpt5/Spt1 ^#^* |
|  | Adult | NB52B, NB54, NB81 and NB97 (Australia) | 6H | 65 | - |  | *Rpt5/Spt1 ^#^* |
| VB9524 × **ND11231** (DH) | Seedling | NB77 (Australia) | 6H | 66 | - |  | *Rpt5/Spt1* |
| **Chevron** × Stander (DH) | Seedling | ND89-19 (USA) | 2HS | 7 | - | Ma et al 2004 | *-* |
|  |  |  | 6HS | 64 | - |  | *Rpt5/Spt1 ^#^* |
| VB9524 × **ND11231** (DH) | Seedling | NB77(Australia) | 2H | 22.80 | *-* | Emebiri et al 2005 | *Rpt3 ^#^* |
|  |  |  | 6H | 75.2 | *QRpt* |  | *Rpt5/Spt1* |
| **OUH602** × Harrington (RIL) | Seedling | 30199013 (USA) | 1H | 10.32 | *Rpt-1H-5-6* | Yun et al 2005 | *Rpt2 ^#^* |
|  |  |  | 3H | 11.50 | *Rpt-3H-4* |  | *Rpt-3H-4* |
|  |  |  | 4H | 9.15-10.33 | *Rpt-4H-5-7* |  | *Rpt7 ^#^* |
| Rolfi × **CI9819** (DH) | Seedling | P7, P8, P40, and P58 (Finland) | 6H | 65 | - | Manninen et al 2000 | *Rpt5/Spt1* |
| Rolfi × **CI9819** (DH) | Seedling | 84-28-1 (USA), 92-46/15 (Canada), UK80-2 (UK), and 27-36 (Finland) | 6H | 60-88 | *Rpt5* | Manninen et al 2006 | *Rpt5/Spt1* |
|  |  |  | 1H | Minor | - |  | *Rpt2* |
|  |  |  | 2H | Minor | - |  | *Rpt3* |
|  |  |  | 3H | Minor | - |  | *Rpt1* |
|  |  |  | 5H | Minor | - |  | *Rpt6* |
|  |  |  | 7H | Minor | - |  | *-* |
| Q21861 × **SM89010** (DH) | Seedling | 15A (USA), ND89-19 (USA) and 0-1 (Canada) | 6H | 84-89 | *-* | Friesen et al 2006 | *Rpt5/Spt1* |
| Alexis × **Sloop** (DH) | Seedling | NB329, NB333, and NB330 (Australia) | 2HS | - | *QNFNBSLR.Al/S-2H* | Lehmensiek et al. 2007 | *QRpts2S* |
|  |  |  | 3HL | - | *QNFNBSLR.Al/S-3H* |  | *Rpt1* |
|  | Adult | NB329, NB333, and NB330 (Australia) | 2HC | 14.5-19.2 | *QNFNBAPR.Al/S-2H* |  | *Rpt3* |
|  |  |  | 3HL | 17.6-30.4 | *QNFNBAPR.Al/S-3H* |  | *Rpt1* |
|  |  |  | 4HC | 10.7-14.0 | *QNFNBAPR.Al/S-4Ha* |  | *Rpt7 ^#^* |
|  |  |  | 4HL | 9.8 | *QNFNBAPR.Al/S-4Hb* |  | *QNFNBAPR.Al/S-4Hb* |
|  |  |  | 7HS | 7.3-8.7 | *QNFNBAPR.Al/S-7Ha* |  | *QNFNBAPR.Al/S-7Ha* |
|  |  |  | 7HL | 11.0 | *QNFNBAPR.Al/S-7Hb* |  | *Rpt4 ^#^* |
| **WI2875-1 (Sloop-sib)** × Alexis (RIL) | Seedling | NB329, NB333, and NB330 (Australia) | 2HS | - | *QNFNBSLR.W/Al-2Ha* |  | *QRpts2S* |
|  |  |  | 2HC | - | *QNFNBSLR.W/Al-2Hb* |  | *Rpt3* |
|  |  |  | 3HL | - | *QNFNBSLR.W/Al-3H* |  | *Rpt1* |
|  | Adult | NB329, NB333, and NB330 (Australia) | 2HC | 8.5-10.7 | *QNFNBAPR.W/Al-2H* |  | *Rpt3* |
|  |  |  | 3HL | 9.6-11.0 | *QNFNBAPR.W/Al-3H* |  | *Rpt1* |
|  |  |  | 4HL | 7.4-12.1 | *QNFNBAPR.W/Al-4H* |  | *QNFNBAPR.Al/S-4Hb ^#^* |
|  |  |  | 5HS | 8.0-12.3 | *QNFNBAPR.W/Al-5H* |  | *-* |
|  |  |  | 7HS | 3.0 | *QNFNBAPR.W/Al-7Ha* |  | *QNFNBAPR.W/Al-7Ha* |
|  |  |  | 7HL | 2.7 | *QNFNBAPR.W/Al-7Hb* |  | *Rpt4 ^#^* |
| **Arapiles** × Franklin (DH) | Seedling | NB329, NB333, and NB330 (Australia) | 2HS | - | *QNFNBSLR.Ar/F-2Ha* |  | *QRpts2S* |
|  |  |  | 2HL | - | *QNFNBSLR.Ar/F-2Hb* |  | *QRpts2L* |
|  |  |  | 3HL | - | *QNFNBSLR.Ar/F-3Ha* |  | *QRpts3La* |
|  |  |  | 3HL | - | *QNFNBSLR.Ar/F-3Hb* |  | *QRpts3Lb* |
|  | Adult | NB329, NB333, and NB330 (Australia) | 1HS | 8.9-12.1 | *QNFNBAPR.Ar/F-1H* |  | *Rpt2 ^#^* |
|  |  |  | 2HS | 16.4 | *QNFNBAPR.Ar/F-2H* |  | *QRpts2S* |
|  |  |  | 7HS | 6.9 | *QNFNBAPR.Ar/F-7H* |  | *QNFNBAPR.W/Al-7Ha* |
| **Rika** × Kombar (DH) | Seedling | 15A (USA) | 6H | 100 | *rpt.r* | Abu Qamar et al 2008 | *Rpt5/Spt1* |
| Rika × **Kombar** (DH) | Seedling | 6A (USA) | 6H | 100 | *rpt.k* |  | *Rpt5/Spt1* |
| CDC Dolly × **TR251** (DH) | Seedling | WRS858 (Canada) | 2H | 8 | *QRptts2* | Grewal et al 2008 | *Rpt3* |
|  |  |  | 4H | 5 | *QRpts4* |  | *Rpt8 ^#^* |
|  |  | WRS1607 and WRS858 (Canada) | 6H | 60-65 | *QRpt6* |  | *Rpt5/Spt1* |
|  | Adult | Natural infection (Melfort, SK, Canada) | 6H | 42-60 | *QRpt6* |  | *Rpt5/Spt1* |
|  |  |  | 3H | 5 | *QRptta3* |  | *QRpts3Lb* |
|  |  |  | 5H | 6 | *QRptta5* |  | *QRptta5* |
|  |  |  | 7H | 6 | *QRpt7* |  | *Rpt4* |
| **CDC Dolly** × TR251 (DH) | Seedling | WRS858 (Canada) | 5H | 7 | *QRptts5* |  | *QRptts5* |
| **M120** × Sep2-72 (RIL) | Seedling | 3010001, 30190005-2, 30199019-1, 30199012-2, and 30199010-3 (-) | 6H-bin2 | 25.0-44.0 | - | St. Pierre et al 2010 | *-* |
|  |  |  | 6H-bin6 | 19.0-48.0 | *-* |  | *Rpt5/Spt1* |
| **Pompadour** × Stirling (DH) | Seedling | NB50, NB52B (Australia) | 3H | 54.2 | *-* | Gupta et al 2010 | *-* |
|  |  | 97NB1, 95NB100, NB81 (Australia) | 6H | 75.8-82.0 | *-* |  | *-* |
| Pompadour × **Stirling** (DH) | Seedling | NB50, NB52B (Australia) | 6H | 18.7-19.8 | *-* |  | *-* |
| **Baudin** × AC Metcalfe (DH) | Seedling | NB50 (Australia) | 2HS | 10 | *-* | Cakir et al 2011 | *-* |
|  | Adult | NB324 (Australia) | 5HS | 6 | *-* |  | *-* |
|  | Seedling/Adult | NB50, NB324, and NB329 (Australia) | 6HS | 6-19 | *-* |  | *Rpt5/Spt1* |
| Baudin × **AC Metcalfe** (DH) | Seedling | NB50 (Australia) | 2HL | 12 | *-* |  | *-* |
|  |  |  | 4HL | 10 | *-* |  | *-* |
|  | Seedling/Adult | NB50 and NB324 (Australia) | 3HS | 10 | *-* |  | *-* |
| **WPG8412** × Stirling (DH) | Seedling | 97NB1, NB73 (Australia) | 6H | 29-86 | *-* | Gupta et al 2011 | *Rpt5/Spt1* |
| **WPG8412** × Pompadour (DH) |  |  | 6H | 23-59 | *-* |  | *Rpt5/Spt1* |
| **Pompadour** × Stirling (DH) |  | 97NB1 (Australia) | 6H | 83 | *-* |  | *Rpt5/Spt1* |
| Pompadour × **Stirling** (DH) |  | NB73 (Australia) | 6H | 83 | *-* |  | *Rpt5/Spt1* |
| **CDC Bold** × TR251 (DH) | Seedling | WRS858 (Canada) | 1H | 12 | *QRptts1.1* | Grewal et al 2012 | *-* |
|  |  | WRS1607 (Canada) | 1H | 8 | *QRptts1.2* |  | *QRptts1.2* |
|  | Adult | Natural Infection (Melfort, SK, Canada) | 5H | 5-12 | *QRptta5.2* |  | *-* |
| CDC Bold × **TR251** (DH) | Seedling/Adult | WRS858 and WRS1607 (Canada), and Natural Infection (Melfort, SK, Canada) | 6H | 32-61 | *QRpt6* |  | *Rpt5/Spt1* |
|  | Adult | Natural Infection (Melfort, SK, Canada) | 2HL | 5 | *QRptta2* |  | *-* |
|  |  |  | 5H | 5-12 | *QRptta5.1* |  | *QRptta5.1* |
| **AT4** × Femina (F_2_) | Seedling | - | 2H | - | *-* | Adawy et al 2013 | *-* |
|  |  |  | 2H | - | *-* |  | *-* |
|  |  |  | 2H | - | *-* |  | *-* |
|  |  |  | 6H | - | *-* |  | *-* |
|  |  |  | LG4 | - | *-* |  | *-* |
|  |  |  | LG4 | - | *-* |  | *-* |
|  |  |  | LG5 | - | *-* |  | *-* |
| Uschi × **HHOR3073** (RIL) | Adult | Natural infection (Quedlinburg and Thüle, Germany) | 2H | 9.4 | *QTL_UH_-2H* | König et al 2013 | *QRpts2L ^#^* |
|  |  |  | 3H | 10.6 | *QTL_UH_-3H* |  | *Rpt-3H-4 ^#^* |
|  |  |  | 5H | 19.0 | *QTL_UH_‐5H-1* |  | *QTL_UH_‐5H-1* |
|  |  |  | 5H | 17.5 | *QTL_UH_-5H-2* |  | *QRptta5.1 ^#^* |
| (Post × Viresa) × **HHOR9484** (RIL) | Adult | Natural infection (Quedlinburg and Thüle, Germany) | 5H | 15.9 | *QTL_PH_-5H-1* |  | *QRptta5 ^#^* |
|  |  |  | 5H | 12.6 | *QTL_PH_-5H-3* |  | *QRptts5* |
|  |  |  | 7H | 22.6 | *QTL_PH_-7H* |  | *QNFNBAPR.Al/S-7Ha ^#^* |
| **(Post × Viresa)** × HHOR9484 (RIL) | Adult | Natural infection (Quedlinburg and Thüle, Germany) | 5H | 34.7 | *QTL_PH_‐5H-2* |  | *QTL_PH_‐5H-2* |
| Uschi × **HHOR3073** (RIL) | Seedling | QLB (Germany) | 7HS | 100 | *Iso_QLB* | König et al 2014 | *QTL_UHs_‐7H* |
|  |  | WvB (Germany) | 3HS | 10.3 | *QTL_UHs_‐3H* |  | *-* |
|  |  |  | 7HS | 32.2 | *QTL_UHs_‐7H* |  | *QTL_UHs_‐7H* |
|  |  | d8_4 (Russia) | 3HS | 57.4 | *QTL_UHs_‐3H‐1* |  | *QTL_UHs_‐3H‐1* |
|  |  |  | 3HS | 12.5 | *QTL_UHs_‐3H‐2* |  | *Rpt-3H-4 ^#^* |
| (Post × Viresa) × **HHOR9484** (RIL) | Seedling | AR (Germany) | 3HL | 77.7 | *QTL_PHs_‐3H* |  | *-* |
|  |  | net1840 (Canada) | 4HL | 48.8 | *QTL_PHs_‐4H* |  | *QTL_PHs_‐4H* |
|  |  |  | 5HS | 12.3 | *QTL_PHs_‐5H* |  | *QRptta5 ^#^* |
| Hector × **Nomini** (F_2_) | Seedling | ND89-19 (USA) | 6H | 90 | *Rpt-Nomini* | O’Boyle et al 2014 | *Rpt5/Spt1* |
| Hector × **CIho2291** (F_2_) | Seedling | ND89-19 (USA) | 6H | 65 | *Rpt-CIho2291* |  | *Rpt5/Spt1* |
| Zernogradsky 813 × **Ranniy 1** (DH) | Seedling | PK4, PP7, PN18, PP5 (Russia) | 1H | 13-20 | *QTL_Afanasenko_1H_50-86** | Afanasenko et al 2015 | *QRptts1.2* |
|  |  | PP6, PK5 (Russia) | 1H | 15-17 | *QTL_Afanasenko_1H_50-86** |  | *QRptts1.2* |
|  |  | PP5 (Russia) | 1H | 20 | *QTL_Afanasenk_1H_96-107** |  | *-* |
|  |  | PN10 (Russia) | 2H | 13 | *QTl_Afanasenk_2H_51-75** |  | *QRptta-2H-57-59* |
|  |  | PL9 (Russia) | 3H | 8 | *QTL_Afanasenko_3H_112-150** |  | *QRptta3* |
|  |  | PK5 (Russia) | 4H | 11 | *QTL_Afanasenko_4H_3-16** |  | *QRptts-4HS* |
|  |  | PN19 (Russia) | 4H | 10 | *QTL_Afanasenk_4H_52-59** |  | *Rpt7* |
|  |  | PN3 (Russia) | 5H | 9 | *QTL_Afanasenko_5H_56-92** |  | *QRptts5* |
|  |  | PP7 (Russia) | 5H | 12 | *QTL_Afanasenk_5H_108-163** |  | *-* |
|  |  | PP1, PP6 (Russia) | 6H | 17-21 | *QTL_Afanasenko_6H_94-126** |  | *AL_QRptt6-2* |
|  |  | PL7 (Russia) | 7H | 11 | *QTL_Afanasenko_7H_61-75** |  | *-* |
| **Hector** × NDB112 (RIL) | Seedling | 15A (USA) | 1HL | 9 | *QTL_Liu_1H_96-100** | Liu et al 2015 | *QRptta-1H-4.11* |
| Hector × **NDB112** (RIL) | Seedling | 15A (USA), NB022 (Australia) | 2HS | 10-13 | *QTL_Liu_2H_0-24** |  | *-* |
|  |  | LDN07Pt5 (USA) | 3HS | 20 | *QTL_Liu_3H_0-2** |  | *NBP_QRptt3-1* |
|  |  | BB06 (Denmark), NB50 (Australia), BrPteres (Brazil) | 3H | 64-73 | *QTL_Liu_3H_68-70** |  | *QRptms3-2 ^#^* |
|  |  | JPT9901 (Japan) | 3HL | 7 | *QTL_Liu_3H_138-144** |  | *QRpts3La* |
|  |  | 0-1 (Canada), LDN07Pt5, ND89-19, 6A (USA), BB06 (Denmark), NB50 (Australia), BrPteres (Brazil) | 3HL | 1-31 | *QTL_Liu_3H_162-170** |  | *QPt.3H-5* |
|  |  | ND89-19 (USA), JPT9901 (Japan), BrPteres (Brazil) | 5HS | 7-18 | *QTL_Liu_5H_28-36** |  | *QRptta-5H-43.76* |
|  |  | 6A (USA), NB50 (Australia) | 5HS | 7-22 | *QTl_Liu_5H_38-46** |  | *Qns-5H.6* |
|  |  | 0-1 (Canada), 15A, LDN07Pt5, ND89-19 (USA), NB022 (Australia) | 6H | 21-34 | *SPN1* |  | *Rpt5/Spt1* |
|  |  | JPT9901 (Japan) | 6H | 21 | *QTL_Liu_6H_54** |  | *QPt.6H-3* |
| CI5791 × **Tifang** (RIL) | Seedling | 15A, 6A (USA), BrPteres (Brazil), BB06 (Denmark) | 3H | 18-28 | *QTL_Koladia_3H-1** | Koladia et al 2017a | *QRptms3-2 ^#^* |
| **CI5791** × Tifang (RIL) | Seedling | 6A (USA) | 1H | 11 | *QTL_Koladia_1H** |  | *-* |
|  |  | JPT0101, JPT9901 (Japan) | 3H | 8.1-23 | *QTL_Koladia_3H-1** |  | *QRptms3-2 ^#^* |
|  |  | 6A (USA) | 3H | 8 | *QTL_Koladia_3H-2** |  | *-* |
|  |  | LDNH04-Ptt-19, Tra-A5, FGOH04Ptt-21, 15A, 6A (USA), JPT0101, JPT9901 (Japan), BrPteres (Brazil), BB06 (Denmark) | 6H | 30-86 | *QTL_Koladia_6H** |  | *Rpt5/Spt1* |
| **Falcon** × Azhul (RIL) | Seedling | 6A (USA) | 2H | 9.49 | *QTL_Islamovic_2H_7.5** | Islamovic et al 2017 | *Qrpts2L.1* |
|  |  | NB50 (Australia) | 2H | 10.78 | *QTL_Islamovic_2H_10.9** |  | *Qrpts2L.2* |
|  |  | NB50 (Australia) | 3H | 13.53 | *QTL_Islamovic_3H_37.4** |  | *Rpt-3H-4* |
|  |  | 0-1, 6A (USA), JPT0101 (Japan), NB50 (Australia) | 4H | 12.26-51.1 | *QTL_Islamovic_4H_31.0-31.2** |  | *Rpt7* |
|  |  | 0-1 (USA), JPT0101 (Japan) | 6H | 6.16-7.6 | *QTL_Islamovic_6H_47.3** |  | *Rpt5/Spt1* |
| Barley Core Collection (Natural) | Seedling | 15A (USA) | 2H | 0.8 | *Qrpts2L.1* | Richards et al 2017 | *QRptma2-3 ^#^* |
|  |  | 15A (USA) | 2H | 1.5 | *Qrpts2L.2* |  | *QRptma2-3 ^#^* |
|  |  | LDNPt19 (USA) | 3H | 2.4 | *QTL_Richards_3H_2.41-2.69** |  | *NBP_QRptt3-1* |
|  |  | 15A, LDNPt19 (USA) | 3H | 0.8-1.3 | *Rpt-3H-4* |  | *Rpt-3H-4* |
|  |  | LDNPt19 (USA) | 3H | 0.9 | *Qrpts3La* |  | *Qrpts3La* |
|  |  | 15A (USA) | 3H | 0.9 | *QRptts-3HL* |  | *QRptts-3HL* |
|  |  | 15A (USA) | 4H | 0.8 | *QRptts-4HS* |  | *QRptts-4HS* |
|  |  | 6A, LDNPt19 (USA) | 4H | 0.8-1.0 | *Qrpts4* |  | *Rpt8* |
|  |  | LDNPt19 (USA) | 5H | 0.8 | *QRptts-5HL.1* |  | *QRptts-5HL.1* |
|  |  | 6A (USA) | 5H | 0.6 | *QRptts-5HL.2* |  | *QTL_UH_‐5H-1* |
|  |  | 15A, 6A, LDNPt19 (USA) | 6H | 1.7- 2.5 | *Rpt5/Spt1/Qrpt6/Qrpts6L* |  | *Rpt5/Spt1* |
|  |  | 6A (USA) | 7H | 0.6 | *QRptts-7HL.1* |  | *QRptts-7HL.1* |
|  |  | 15A, 6A, LDNPt19 (USA) | 7H | 0.8-1.0 | *QTL_UHs_‐7H* |  | *QTL_UHs_‐7H* |
| **H602** × Haruna Nijo (DH) | Seedling | Japanese Isolate | 3H | - | *-* | Hisano et al 2017 | *-* |
|  |  |  | 3H | - | *-* |  | *-* |
|  |  |  | 6H | - | *-* |  | *Rpt5/Spt1* |
| **Arve** × Lavrans (DH) | Seedling | 5050B, 6949B (Norway) | 4H | 12-16.5 | *AL_QRptt4-1* | Wonneberger et al 2017a | *Rpt7* |
| Arve × **Lavrans** (DH) | Seedling | 5050B, 6949B, LR9 (Norway) | 5H | 11.5-14.8 | *AL_QRptt5-1* |  | *QRptts5* |
|  |  | 5050B, LR9 (Norway) | 6H | 11.6-14.0 | *AL_QRptt6-1* |  | *AL_QRptt6-1* |
|  | Adult/Seedling | 5050B, 6949B, LR9 (Norway) | 3H | 10.8 | *AL_QRptt3-1* |  | *QRptta3* |
|  |  |  | 5H | 15.5-47.6 | *AL_QRptt5-2* |  | *QPt.5H-3* |
|  |  |  | 7H | 11.1-12.1 | *AL_QRptt7-2* |  | *SFNB-7H-34.82* |
|  | Adult |  | 6H | 9.7-14.8 | *AL_QRptt6-2* |  | *AL_QRptt6-2* |
|  |  |  | 6H | 10.7-11.0 | *AL_QRptt6-3* |  | *AL_QRptt6-3* |
|  |  |  | 7H | 11.9 | *AL_QRptt7-1* |  | *QNFNBAPR.Al/S-7Ha ^#^* |
| Nordic Barley Panel (Natural) | Seedling | 6949B (Norway) | 2H | 10 | *NBP_QRptt2-1* | Wonneberger et al 2017b | *SFNB-2H-8-10* |
|  |  | LR9 (Norway) | 4H | 8 | *NBP_QRptt4-1* |  | *QRptts-4HS* |
|  |  |  | 4H | 7 | *NBP_QRptt4-2* |  | *Rpt7* |
|  |  |  | 7H | 8 | *NBP_QRptt7-3* |  | *QTL_UHs_‐7H ^#^* |
|  | Seedling/Adult | LR9, 5050B, 6949B (Norway) | 3H | 6-15 | *NBP_QRptt3-2* |  | *QRptms3-2 ^#^* |
|  |  | 5050B, 6949B (Norway) | 6H | 9-10 | *NBP_QRptt6-1* |  | *Rpt5/Spt1* |
|  | Adult |  | 1H | 6-14 | *NBP_QRptt1-1* |  | *NBP_QRptt1-1* |
|  |  |  | 1H | 6 | *NBP_QRptt1-2* |  | *NBP_QRPtt1-2* |
|  |  |  | 3H | 5 | *QTL_UHs_‐3H-1* |  | *NBP_QRptt3-1* |
|  |  |  | 5H | 6-11 | *NBP_QRptt5-1* |  | *QRptta5 ^#^* |
|  |  |  | 5H | 7-10 | *NBP_QRptt5-2* |  | *QPt.5H-3* |
|  |  |  | 7H | 10 | *NBP_QRptt7-1* |  | *SFNB-7H-34.82* |
|  |  |  | 7H | 6 | *NBP_QRptt7-2* |  | *Rpt4* |
| Halle Exotic Barley 25 (NAM) | Adult (RT) | Natural infection (Quedlinburg, Germany) | 1H | 2.33 | *QPt.1H-1* | Vatter et al 2017 | *QRptts1.2* |
|  |  |  | 2H | 9.23 | *QPt.2H-1* |  | *QRpts2S* |
|  |  |  | 3H | 0.87 | *QPt.3H-1* |  | *QTL_UHs_‐3H-1* |
|  |  |  | 3H | 8.64 | *QPt.3H-3* |  | *QRptms3-2 ^#^* |
|  |  |  | 4H | 1.04 | *QPt.4H-1* |  | *QRptts-4HS* |
|  |  |  | 4H | 0.85 | *QPt.4H-3* |  | *QPt.4H-3* |
|  |  |  | 4H | 6.64 | *QPt.4H-4* |  | *QNFNBAPR.Al/S-4Hb ^#^* |
|  |  |  | 5H | 1.63 | *QPt.5H-1* |  | *QTL_UH_‐5H-1 ^#^* |
|  |  |  | 6H | 0.08 | *QPt.6H-1* |  | *Rpt5/Spt1* |
|  |  |  | 6H | 1.37 | *QPt.6H-3* |  | *QPt.6H-3* |
|  |  |  | 7H | 0.10 | *QPt.7H-3* |  | *QTL_UHs_‐7H ^#^* |
|  | Adult (AO) | Natural infection (Quedlinburg, Germany) | 2H | 14.88 | *QPt.2H-2* |  | *QRpts2S* |
|  |  |  | 2H | 0.07 | *QPt.2H-3* |  | *Rpt3* |
|  |  |  | 3H | 0.78 | *QPt.3H-2* |  | *QTL_UHs_‐3H-1* |
|  |  |  | 3H | 0.04 | *QPt.3H-4* |  | *QRpts3La* |
|  |  |  | 3H | 0.12 | *QPt.3H-5* |  | *QPt.3H-5* |
|  |  |  | 4H | 0.36 | *QPt.4H-2* |  | *QRptts-4HS* |
|  |  |  | 4H | 6.50 | *QPt.4H-5* |  | *QNFNBAPR.Al/S-4Hb ^#^* |
|  |  |  | 5H | 1.50 | *QPt.5H-2* |  | *QRptts5* |
|  |  |  | 5H | 0.41 | *QPt.5H-3* |  | *QPt.5H-3* |
|  |  |  | 6H | 0.39 | *QPt.6H-2* |  | *Rpt5/Spt1* |
|  |  |  | 6H | 0.04 | *QPt.6H-4* |  | *AL_QRptt6-2* |
|  |  |  | 7H | 3.65 | *QPt.7H-1* |  | *QNFNBAPR.Al/S-7Ha ^#^* |
|  |  |  | 7H | 1.54 | *QPt.7H-2* |  | *SFNB-7H-34.82* |
| **UVC8** × Erica (DH) | Adult | NB50 (Australia) | 3H | 6.6 | *QTL_Martin_3H_59.5-62.9** | Martin et al 2018 | *SFNB-3H-78.53* |
|  |  | Natural Infection (Caledon, South Africa) | 3H | 5.0-7.5 | *QTL_Martin_3H_132.3** |  | *Rpt1 ^#^* |
|  |  | NB73 (Australia) | 4H | 2.9 | *QTL_Martin_4H_74.6-77.0** |  | *QPt.4H-3* |
|  |  | Natural Infection (Caledon, South Africa) | 4H | 4.9-5.6 | *QTL_Martin_4H_110.3-113.1** |  | *-* |
|  |  | NB73, NB50 (Australia), Natural Infection (Caledon, South Africa) | 6H | *16.6-55.0* | *QTL_Martin_6H_67.4-70.5** |  | *Rpt5/Spt1* |
|  |  | NB73, NB85, NB50 (Australia) | 7H | *5.8-8.8* | *QTL_Martin_7H_175.7-176.8** |  | *QTL_UHs_‐7H ^#^* |
| UVC8 × **Erica** (DH) | Adult | NB73 (Australia) | 1H | *3.3-11.6* | *QTL_Martin_1H_19.4-25.7** |  | *-* |
|  |  | Natural Infection (Caledon, South Africa) | 3H | *7.6* | *QTL_Martin_3H_148.5-150.2** |  | *QPt.3H-5* |
|  |  |  | 5H | *8.4-8.9* | *QTL_Martin_5H_91.1-92.3** |  | *QTL_UH_‐5H-1 ^#^* |
| ICARDA AM-2014 Panel (Natural) | Seedling | Ptt19 (USA) | 1H | 3.9 | *QRptts-1H-92-93* | Amezrou et al 2018 | *QRptts1.2* |
|  |  |  | 4H | 3.5 | *QRptts-4H-97.66* |  | *QRptts-4H-97.66* |
|  |  |  | 5H | 3.7 | *QRptts-5H-80.35* |  | *QRptts-5H-80.35* |
|  |  |  | 5H | 3.8 | *QRptts-5H-130.03* |  | *QTL_PH_‐5H-2 ^#^* |
|  |  |  | 6H | 3.5 | *QRptts-6H-78.4* |  | *AL_QRptt6-1* |
|  |  |  | 6H | 3.6 | *QRptts-6H-98.55* |  | *AL_QRptt6-2* |
|  |  |  | 7H | 3.8 | *QRptts-7H-74.29* |  | *QRptts-7H-74.29* |
|  |  | TD10 (USA) | 2H | 6.41 | *QRptts-2H-7.44* |  | *SFNB-2H-8-10* |
|  | Adult/Seedling | TD10 (USA), Natural Infection (Sidi El Ayedi, Morocco) | 2H | 3.7 | *QRptt.2H-132.15* |  | *QRptma2-3 ^#^* |
|  |  | Ptt19 (USA), Natural Infection (Allal Tazi, Sidi El Ayedi, and IAV-HII, Morocco) | 6H | 3.63-5.1 | *QRptt.6H-54-55* |  | *Rpt5/Spt1* |
|  | Adult | Natural Infection (Sidi El Ayedi, Morocco) | 1H | 3.42 | *QRptta-1H-4.11* |  | *QRptta-1H-4.11* |
|  |  |  | 7H | 3.57 | *QRptta-7H-42.28* |  | *QRptta-7H-42.28* |
|  |  | Natural Infection (Jemma Shiam, Morocco) | 1H | 3.39 | *QRptta-1H-125.99* |  | *NBP_QRPtt1-2* |
|  |  |  | 2H | 6.41 | *QRptta-2H-40.79* |  | *SFNB-2H-38.03* |
|  |  |  | 5H | 3.52 | *QRptta-5H-143.4* |  | *SFNB-5H-155.13* |
|  |  |  | 7H | 3.43 | *QRptta-7H-0.39* |  | *QNFNBAPR.Al/S-7Ha ^#^* |
|  |  | Natural Infection (IAV-HII, Morocco) | 2H | 5.43 | *QRptta-2H-92.21* |  | *QRptma2-3 ^#^* |
|  |  |  | 2H | 4.09 | *QRptta-2H-114-117* |  | *QRptma2-3 ^#^* |
|  |  |  | 3H | 3.86 | *QRptta-3H-118.30* |  | *QRptta3* |
|  |  |  | 4H | 4.42 | *QRptta-4H-81.57* |  | *QRptta-4H-81.57* |
|  |  |  | 5H | 3.51 | *QRptta-5H-43.76* |  | *QRptta-5H-43.76* |
|  |  |  | 6H | 3.44 | *QRptta-6H-35.62* |  | *SFNB-6H-33.74* |
|  |  |  | 6H | 4.17 | *QRptta-6H-49.79* |  | *Rpt5/Spt1* |
|  |  |  | 7H | 4.26 | *QRptta-7H-23.02* |  | *QRptta-7H-23.02* |
|  |  | Natural Infection (Marchouch, Morocco) | 2H | 3.78 | *QRptta-2H-126.77* |  | *QRptma2-3 ^#^* |
|  |  |  | 3H | 3.64 | *QRptta-3H-144.65* |  | *QPt.3H-5* |
|  |  |  | 5H | 3.42 | *QRptta-5H-139.38* |  | *SFNB-5H-155.13* |
|  |  | Natural Infection (Marchouch and IAV-HIII, Morocco) | 2H | 4.00-4.08 | *QRptta-2H-57-59* |  | *Rpt3* |
|  |  | Natural Infection (Allal Tazi, Marchouch and Sidi El Ayedi, Morocco) | 2H | 3.37-3.61 | *QRptta-2H-143.13* |  | *QRptma2-3 ^#^* |
|  |  | Natural Infection (AVI-HII and Jemma Shiam, Morocco) | 3H | 3.37-7.01 | *QRptta-3H-154-155* |  | *Qrptts-3HL* |
|  |  | Natural Infection (Allal Tazi and Sidi El Ayedi, Morocco) | 5H | 3.35-3.41 | *QRptta-5H-160.49* |  | *QPt.5H-3* |
| Ethiopian, ICARDA and NDSU | Seedling | ND89-19 (USA) | 2H | 6.8 | *Qnfnb-2H.1* | Daba et al 2019 | *QRptma2-3 ^#^* |
| Barley Panel (Hybrid) |  |  | 4H | 6.5 | *Qnfnb-4H.1* |  | *QRptts-4HS* |
|  |  |  | 4H | 8.4 | *Qnfnb-4H.2* |  | *QRptts-4H-97.66* |
|  |  |  | 6H | 6.6 | *Qnfnb-6H.1* |  | *Rpt5/Spt1* |
|  |  |  | 6H | 7.5 | *Qnfnb-6H.2* |  | *Rpt5/Spt1* |
|  |  |  | 6H | 7.8 | *Qnfnb-6H.3* |  | *Rpt5/Spt1* |
|  |  |  | 6H | 8.8 | *Qnfnb-6H.4* |  | *Rpt5/Spt1* |
|  | Adult | Natural Infection (Bekoji and Koffele, Ethiopia) | 1H | 11.7-26.0 | *Qns-1H* |  | *NBP_QRPtt1-2* |
|  |  |  | 2H | 16.2-22.9 | *Qns-2H.1* |  | *Rpt3* |
|  |  |  | 2H | 11.5-21.8 | *Qns-2H.2* |  | *Rpt3* |
|  |  |  | 2H | 15.3 | *Qns-2H.3* |  | *Qns-2H.3* |
|  |  |  | 3H | - | *Qns-3H.1* |  | *QTL_UHs_‐3H-1* |
|  |  |  | 3H | 15.6 | *Qns-3H.2* |  | *QTL_UH_-3H* |
|  |  |  | 3H | 11.6-22.1 | *Qns-3H.3* |  | *QRptms3-2 ^#^* |
|  |  |  | 3H | 12.3-18.7 | *Qns-3H.4* |  | *QRpts3La* |
|  |  |  | 3H | 13.0-24.1 | *Qns-3H.5* |  | *Qrptts-3HL* |
|  |  |  | 4H | - | *Qns-4H.1* |  | *SFNB-4H-36.37* |
|  |  |  | 4H | 11.6-22.8 | *Qns-4H.2* |  | *Rpt7* |
|  |  |  | 4H | - | *Qns-4H.3* |  | *Rpt7* |
|  |  |  | 5H | 12.3-22.6 | *Qns-5H.1* |  | *Qns-5H.1* |
|  |  |  | 5H | 16.6 | *Qns-5H.2* |  | *Qns-5H.2* |
|  |  |  | 5H | 12.3-20.0 | *Qns-5H.3* |  | *Qns-5H.3* |
|  |  |  | 5H | 16.4-22.7 | *Qns-5H.4* |  | *Qns-5H.4* |
|  |  |  | 5H | 12.4-20.0 | *Qns-5H.5* |  | *Qns-5H.5* |
|  |  |  | 5H | 11.4-22.2 | *Qns-5H.6* |  | *Qns-5H.6* |
|  |  |  | 5H | 11.7-19.8 | *Qns-5H.7* |  | *QPt.5H-3* |
|  |  |  | 6H | 11.2-19.7 | *Qns-6H.1* |  | *SFNB-6H-5.4* |
|  |  |  | 6H | 10.7-13.6 | *Qns-6H.2* |  | *SFNB-6H-5.4* |
|  |  |  | 6H | - | *Qns-6H.3* |  | *SFNB-6H-5.4* |
|  |  |  | 6H | - | *Qns-6H.4* |  | *SFNB-6H-33.74* |
|  |  |  | 6H | 12.0-14.6 | *Qns-6H.5* |  | *Rpt5/Spt1* |
|  |  |  | 7H | 11.9-21.8 | *Qns-7H.1* |  | *QNFNBAPR.Al/S-7Ha ^#^* |
|  |  |  | 7H | - | *Qns-7H.2* |  | *Rpt4* |
| **Canadian Lake Shore** × Harrington (DH) | Seedling | Len7, Ps31, Pr11, Vol1 (Russia), Bel1 (Belarus), G5 (German), Can11 (Canada), SA7 (South Africa) | 3H | 26-78 | *qPttCLS* | Dinglasan et al 2019 | *Rpt-3H-4 ^#^* |
| Siberian barley panel (Artificial) | Seedling | P3.4.0, K5.1 (Russia) | 1H | *-* | *QTl_Rozanova_1h_57.3-62.8** | Rozanova et al 2019 | *-* |
|  |  | S10.2 (Finland) | 2H | *-* | *QTL_Rozanova_2H_23.2-23.8** |  | *Rpt3* |
|  |  | P3.4.0 (Russia) | 2H | *-* | *QTL_Rozanova_2H_71.0-74.1** |  | *Qns-2H.3* |
|  |  | P3.4.0 (Russia) | 3H | *-* | *QTL_Rozanova_3H_12.1-17.4** |  | *QTL_UHs_‐3H-1* |
|  |  | A2.6.0 (Russia) | 3H | - | *QTL_Rozanova_3H_50.9-54.8** |  | *QRptms3-2 ^#^* |
|  |  | K5.1 (Russia) | 3H | - | *QTL_Rozanova_3H_135.6-137.5** |  | *Qrptts-3HL* |
|  |  | P3.4.0, K5.1, S10.2 (Finland) | 6H | - | *QTL_Rozanova_6H_52.6-55.4** |  | *Rpt5/Spt1* |

* Resistant parental lines are indicated by bold type; DH, double haploid; F_2_, second generation of selfing; RIL, recombinant inbred line; BC_1_F_2_^,^ second generation of backcross; NAM, nested association mapping population; Artificial; breeding line panel; Natural; landraces and wild barley panel; Hybrid, artificial and natural barley panel.

† Stage at which resistance is active; RT, reaction type phenotyping; AO, average ordinate phenotyping.

‡ County of origin given if isolate, location of infection given if natural infection

§ QTL effects containing ranges mean that the experiments were performed for multiple locations or multiple isolates and the effects for individual treatments fall into this range.

‖ References may contain more than one population; ^1^Publication also reported this locus.

¶ Inferred locus designation using respective references of colocalising loci using BarleyMap (Cantalapiedra et al., 2015) or T3/Barley; earliest published designation given if locus was not previously designated; ^#^ not confirmed.

-, no information is available for this entry.

Updated and modified from Liu et al. (2011).
