# Supplementary material for: Research advances in the Pyrenophora teres–barley interaction
Source: Mol Plant Pathol. 2019 Dec 13;21(2):272–88. doi: 10.1111/mpp.12896 (PMC6988421; doi:10.1111/mpp.12896)
Supplement: Supplementary file 2 — Table S2 Studies mapping barley resistance/susceptibility genes to Pyrenophora teres f. maculata. Different populations are indicated by alternating grey scale with the parent contributing the resistance allele in bold. Plant stage indicates whether the resistance is effective at the seedling or adult stage. If a single genotype isolate is used this is indicated by the name and country of origin, whereas natural infection is indicated by specific location. Barley chromosome, phenotypic variation and designation of each locus are displayed if available from the corresponding reference. The inferred locus designation is reported based on markers obtained from the relevant publication and collapsed into loci using a maximum distance of 10 Mb of the Morex reference genome (Mascher et al., 2017) using BarleyMap (Cantalapiedra et al., 2015) or T3/Barley (Fig. 1). [file MPP-21-272-s002.docx]

**Supplemental Table S2.** Studies mapping barley resistance/susceptibility genes to *Pyrenophora teres* f. *maculata.* Different population are indicated by alternating grey scale with parent contributing the resistance allele in bold. Plant stage indicates whether the resistance is active at the seedling or adult stage. If a single genotype isolate is used this is indicated by the name and country of origin, whereas natural infection is indicated by specific location. Barley chromosome, phenotypic variation and designation of each locus is displayed if available from the corresponding reference. The inferred locus designation is reported based on markers obtained from the relevant publication, and collapsed into loci using a maximum distance of 10 Mb of the Morex reference genome (Mascher et al., 2017) using BarleyMap (Cantalapiedra et al., 2015) or T3/Barley (Supplemental Table 3), inability to genetically separate in mapping or article discussion if possible.

| Population (type) * | Plant Stage † | Isolate (origin) ‡ | Chr. Location | R^2^ (%) § | QTL Designation | References ‖ | Inferred Locus Designation ¶ |
| --- | --- | --- | --- | --- | --- | --- | --- |
| **Léger** × CI9831 (DH) | Seedling | WRS857 (Canada) | - | - | *-* | Ho et al 1996 | *Rpt3 ^#^* |
| **Léger** × CI9831 (DH) | Seedling | WRS857 (Canada) | 2H | - | - | Molnar et al 2000 | *Rpt3 ^#^* |
|  |  |  | LG1 | - | *-* |  | *-* |
|  |  |  | LG3 | - | *-* |  | *-* |
| **Galleon** × Haruna Nijo (DH) | Seedling | 43/96/1, 49/96/9, 49/96/10, 50/96/9, and 10/97 (Australia) | 7H | 27-74 | *Rpt4* | Williams et al 1999 | *Rpt4* |
| **CI9214** × Stirling (DH) | Seedling | - | 7H | 52 | *Rpt4* | Williams et al 2003 | *Rpt4* |
| **Keel** × Gairdner (DH) | Seedling | - | 7H | 46 | *Rpt4* |  | *Rpt4* |
| **Tilga** × Tantangara (DH) | Seedling | - | 7H | 27 | *Rpt4* |  | *Rpt4* |
| **Chebec** × Harrington (DH) | Seedling | - | 7H | 74 | *Rpt4* |  | *Rpt4* |
| **VB9104** × Dash (DH) | Seedling | - | 7H | 9 | - |  | *-* |
|  |  |  | 1H | Minor | - |  | *-* |
|  |  |  | 3H | Minor | - |  | *-* |
| **Galleon** × Haruna Nijo (DH) | Adult | - | 7H | 18 | - |  | *-* |
|  |  |  | 7H | 20 | *Rpt4* |  | *Rpt4* |
|  |  |  | 7H | 27 | - |  | *-* |
|  |  |  | 5H | 19 | - |  | *-* |
|  |  |  | 4H | 8 | - |  | *-* |
| **VB9104** × Dash (DH) | Adult | - | 7H | 17 | - |  | *-* |
|  |  |  | 5H | 9 | - |  | *-* |
|  |  |  | 4H | 10 | - |  | *-* |
| **CI9214** × Stirling (DH) | Adult | - | 7H | 35 | *Rpt4* |  | *Rpt4* |
| **Keel** × Gairdner (DH) | Adult | - | 7H | 32 | *Rpt4* |  | *Rpt4* |
| **Tilga** × Tantangara (DH) | Adult | - | 7H | 7 | *Rpt4* |  | *Rpt4* |
| **Q21861** × SM89010 (DH) | Seedling | NZKF2 (New Zealand) | 4H | 64 | *QTL_Friesen* | Friesen et al 2006 | *Rpt8* |
| Rolfi × **CI9819** (DH) | Seedling | P1332 and P1333 (Finland) | 5H | 65-84 | *Rpt6* | Manninen et al 2006 | *Rpt6* |
| CDC Dolly × **TR251** (DH) | Seedling | WRS857 (Canada) | 4H | 21 | *QRpts4* | Grewal et al 2008 | *Rpt7 ^#^* |
|  |  |  | 7H | 13 | *QRpt7* |  | *Rpt4 ^#^* |
|  |  |  | 6H | 8 | *QRpt6* |  | *Rpt5/Spt1* |
| **Baudin** × AC Metcalfe (DH) | Seedling/Adult | 95NB104, 95NB117, WAC11160, and NB320 (Australia) | 6HS | 9-47 | *-* | Cakir et al 2011 | *-* |
|  | Adult | NB320 (Australia) | 2HL | 8 | *-* |  | *-* |
|  |  |  | 3HS | 8 | *-* |  | *-* |
|  |  |  | 7HL | 5 | *-* |  | *Rpt4 ^#^* |
| CDC Bold × **TR251** (DH) | Seedling | WRS857 (Canada) | 1H | 14 | *QRptms1* | Grewal et al 2012 | *-* |
|  |  |  | 4H | 13 | *QRptms4* |  | *-* |
|  |  |  | 6H | 22 | *QRptms6* |  | *-* |
|  |  | LO233 (Canada) | 2HL | 18 | *QRptms2* |  | *-* |
| Barley Core Collection (Natural) | Seedling | SG1 (Australia) | 1H | 0.1 | *QTL_Tamang_1H_50** | Tamang et al 2015 | *NBP_QRptt1-1* |
|  |  | DEN2.6 (Denmark) | 1H | 1.5 | *QTL_Tamang_1H_82.5** |  | *-* |
|  |  | SG1 (Australia) | 1H | 0.8 | *QTL_Tamang_1H_94.45** |  | *QPt.1H-1* |
|  |  | FGO (USA) | 1H | 3.3 | *QTL_Tamang_1H_114.3** |  | *-* |
|  |  | NZKF2 (New Zealand) | 2H | 0.4 | *QTL_Tamang_2H_23.76** |  | *SFNB-2H-8-10* |
|  |  | DEN2.6 (Denmark), NZKF2 (New Zealand), SG1 (Australia), FGO (USA) | 2H | 1.8-8.6 | *QTL_Tamang_2H_65.71-69.55** |  | *QRpts2Sb* |
|  |  | FGO (USA) | 2H | 0.03 | *QTL_Tamang_2H_137.44** |  | *QRptma2-3 ^#^* |
|  |  | DEN2.6 (Denmark), NZKF2 (New Zealand) | 3H | 7.7-8.4 | *QTL_Tamang_3H_53.42** |  | *Rpt-3H-4 ^#^* |
|  |  | DEN2.6 (Denmark) | 3H | 5.2 | *QTL_Tamang_3H_65.16** |  | *-* |
|  |  | SG1 (Australia) | 3H | 4.2 | *QTL_Tamang_3H_88.17** |  | *QRpts3La* |
|  |  | NZKF2, FGO (USA) | 3H | 0.13-0.2 | *QTL_Tamang_3H_99.26-103.86** |  | *Rpt1 ^#^* |
|  |  | DEN2.6 (Denmark), NZKF2 (New Zealand) | 3H | 4.8-5.3 | *QTL_Tamang_3H_150.19-154.47** |  | *QRptts-3HL* |
|  |  | SG1 (Australia) | 4H | 0.5 | *QTL_Tamang_4H_32.43** |  | *SFNB-4H-36.37* |
|  |  | SG1 (Australia) | 4H | 0.3 | *QTL_Tamang_4H_47.17** |  | *Rpt7* |
|  |  | DEN2.6 (Denmark), NZKF2 (New Zealand) | 4H | 14.8-16.0 | *QRpts4* |  | *Rpt7* |
|  |  | DEN2.6 (Denmark), NZKF2 (New Zealand), FGO (USA) | 4H | 0.4-9.0 | *QTL (Rpt8)* |  | *Rpt8* |
|  |  | NZKF2 (New Zealand) | 4H | 11.9 | *QTL_Tamang_4H_96.6-103.58** |  | *QPt.4H-4* |
|  |  | SG1 (Australia), FGO (USA) | 5H | 0.3-0.8 | *Rpt6* |  | *Rpt6* |
|  |  | DEN2.6 (Denmark), NZKF2 (New Zealand) | 5H | 0.1-0.3 | *QTL_Tamang_5H_111.56** |  | *-* |
|  |  | DEN2.6 (Denmark), NZKF2 (New Zealand), FGO (USA) | 6H | 1.8-4.1 | *QRpt6* |  | *Rpt5/Spt1* |
|  |  | SG1 (Australia) | 6H | 0.0 | *QTL_Tamang_6H_101.83** |  | *AL_QRptt6-1* |
|  |  | DEN2.6 (Denmark) | 7H | 1.3 | *QTL_Tamang_7H_0** |  | *QNFNBAPR.Al/S-7Ha ^#^* |
|  |  | FGO (USA) | 7H | 1.9 | *QRpt4/Rpt4* |  | *QNFNBAPR.Ar/F-7H ^#^* |
|  |  | NZKF2 (New Zealand) | 7H | 0.3 | *QTL_Tamang_7H_78.07** |  | *QRptm7-3 ^#^* |
|  |  | FGO (USA) | 7H | 0.9 | *QTL_Tamang_7H_109** |  | *Rpt4* |
|  |  | NZKF2 (New Zealand), FGO (USA) | 7H | 0.6-1.9 | *QRpt7/Rpt7* |  | *QRptm7-6 ^#^* |
|  |  | NZKF2 (New Zealand) | 7H | 1.9 | *QTL_Tamang_7H_145.68-150.36** |  | *QTL_UHs_‐7H ^#^* |
| Northern Region Barley Breeding | Seedling | SNB331 and SNB320 (Australia) | 2H | 3.0 | *QRptms2-4* | Wang et al 2015 | *-* |
| Program of Australia (Artificial) |  |  | 3H | 2.6 | *QRptms3-2* |  | *QRptms3-2* |
|  | Adult | SNB331 and SNB320 (Australia) | 2H | 1.3 | *QRptma2-3* |  | *QRptma2-3* |
|  |  |  | 5H | 1.5 | *QRptma5-2* |  | *-* |
|  |  |  | 5H | 2.5 | *QRptma5-3* |  | *-* |
|  |  |  | 5H | 2.6 | *QRptma5-5* |  | *-* |
|  |  |  | 6H | 1.4 | *QRptma6-4* |  | *QRptma6-4* |
|  | Seedling/Adult | SNB331 and SNB320 (Australia) | 1H | 1.6-2.6 | *QRptm1-1* |  | *-* |
|  |  |  | 2H | 2.5-2.8 | *QRptm2-1* |  | *-* |
|  |  |  | 2H | 2.6-3.4 | *QRptm2-2* |  | *-* |
|  |  |  | 3H | 1.5 | *QRptm3-1* |  | *-* |
|  |  |  | 3H | 2.7 | *QRptm3-3* |  | *-* |
|  |  |  | 3H | 2.4-3.4 | *QRptm3-4* |  | *-* |
|  |  |  | 4H | 2.7 | *QRptm4-1* |  | *-* |
|  |  |  | 4H | 2.5 | *QRptm4-2* |  | *-* |
|  |  |  | 5H | 2.5-3.3 | *QRptm5-1* |  | *-* |
|  |  |  | 5H | 1.7 | *QRptm5-4* |  | *-* |
|  |  |  | 5H | 2.6 | *QRptm5-6* |  | *QRptm5-6* |
|  |  |  | 6H | 2.8 | *QRptm6-1* |  | *-* |
|  |  |  | 6H | 2.5 | *QRptm6-2* |  | *Rpt5/Spt1* |
|  |  |  | 6H | 1.5 | *QRptm6-3* |  | *-* |
|  |  |  | 7H | 3.1 | *QRptm7-1* |  | *-* |
|  |  |  | 7H | 2.0 | *QRptm7-2* |  | *-* |
|  |  |  | 7H | 2.5-3.1 | *QRptm7-3* |  | *QRptm7-3 ^#^* |
|  |  |  | 7H | 1.8-3.8 | *QRptm7-4* |  | *Rpt4 ^#^* |
|  |  |  | 7H | 1.5-3.0 | *QRptm7-5* |  | *Rpt4 ^#^* |
|  |  |  | 7H | 2.4-2.8 | *QRptm7-6* |  | *QRptm7-6 ^#^* |
|  |  |  | 7H | 3.8-4.4 | *QRptm7-7* |  | *-* |
|  |  |  | 7H | 1.7-3.9 | *QRptm7-8* |  | *-* |
| Upper Midwest Breeding | Seedling | SFNB-MT09 (USA) | 2HS | 3.2-6.5 | *SFNB-2H-8-10* | Burlakoti et al 2017 | *SFNB-2H-8-10* |
| Programs (Artificial) |  |  | 2HS | 1.5 | *SFNB-2H-38.03* |  | *SFNB-2H-38.03* |
|  |  |  | 3HL | 1.7 | *SFNB-3H-58.64* |  | *QRptms3-2 ^#^* |
|  |  |  | 3HL | 1..5 | *SFNB-3H-78.53* |  | *SFNB-3H-78.53* |
|  |  |  | 3HL | 1.4-4.4 | *SFNB-3H-91.88* |  | *QRpts3La* |
|  |  |  | 3HL | 1.8-4.3 | *SFNB-3H-117.1* |  | *Rpt1 ^#^* |
|  |  |  | 4HS | 3.5 | *SFNB-4H-36.37* |  | *SFNB-4H-36.37* |
|  |  |  | 5HL | 1.7 | *SFNB-5H-155.13* |  | *SFNB-5H-155.13* |
|  |  |  | 6HS | 1.5-3.5 | *SFNB-6H-5.4* |  | *SFNB-6H-5.4* |
|  |  |  | 6HS | 1.5 | *SFNB-6H-33.74* |  | *SFNB-6H-33.74* |
|  |  |  | 7HS | 1.5-3.8 | *SFNB-7H-34.82* |  | *SFNB-7H-34.82* |
| Ethiopian, ICARDA and NDSU | Seedling | ND111 (USA) | 6H | 10.2 | *Qsfnb-6H* | Daba et al 2019 | *Rpt5/Spt1* |
| Barley Panel (Hybrid) | Adult | Natural Infection (Bekoji and Koffele, Ethiopia) | 1H | 11.7-26.0 | *Qns-1H* |  | *NBP_QRPtt1-2* |
|  |  |  | 2H | 16.2-22.9 | *Qns-2H.1* |  | *QRptta-2H-57-59* |
|  |  |  | 2H | 11.5-21.8 | *Qns-2H.2* |  | *QRptta-2H-57-59* |
|  |  |  | 2H | 15.3 | *Qns-2H.3* |  | *QRpts2Sb* |
|  |  |  | 3H | - | *Qns-3H.1* |  | *QTL_UHs_‐3H-1* |
|  |  |  | 3H | 15.6 | *Qns-3H.2* |  | *QTL_UH_-3H* |
|  |  |  | 3H | 11.6-22.1 | *Qns-3H.3* |  | *QTL_UH_-3H* |
|  |  |  | 3H | 12.3-18.7 | *Qns-3H.4* |  | *QRpts3La* |
|  |  |  | 3H | 13.0-24.1 | *Qns-3H.5* |  | *Qrptts-3HL* |
|  |  |  | 4H | - | *Qns-4H.1* |  | *SFNB-4H-36.37* |
|  |  |  | 4H | 11.6-22.8 | *Qns-4H.2* |  | *Rtp8* |
|  |  |  | 4H | - | *Qns-4H.3* |  | *Rpt8* |
|  |  |  | 5H | 12.3-22.6 | *Qns-5H.1* |  | *Qns-5H.1* |
|  |  |  | 5H | 16.6 | *Qns-5H.2* |  | *Qns-5H.2* |
|  |  |  | 5H | 12.3-20.0 | *Qns-5H.3* |  | *Qns-5H.3* |
|  |  |  | 5H | 16.4-22.7 | *Qns-5H.4* |  | *Qns-5H.4* |
|  |  |  | 5H | 12.4-20.0 | *Qns-5H.5* |  | *Qns-5H.5* |
|  |  |  | 5H | 11.4-22.2 | *Qns-5H.6* |  | *Qns-5H.6* |
|  |  |  | 5H | 11.7-19.8 | *Qns-5H.7* |  | *QPt.5H-3* |
|  |  |  | 6H | 11.2-19.7 | *Qns-6H.1* |  | *SFNB-6H-5.4* |
|  |  |  | 6H | 10.7-13.6 | *Qns-6H.2* |  | *SFNB-6H-5.4* |
|  |  |  | 6H | - | *Qns-6H.3* |  | *SFNB-6H-5.4* |
|  |  |  | 6H | - | *Qns-6H.4* |  | *SFNB-6H-33.74* |
|  |  |  | 6H | 12.0-14.6 | *Qns-6H.5* |  | *Rpt5/Spt1* |
|  |  |  | 7H | 11.9-21.8 | *Qns-7H.1* |  | *QNFNBAPR.Al/S-7Ha ^#^* |
|  |  |  | 7H | - | *Qns-7H.2* |  | *Rpt4* |
| Tradition × **PI67381** (RIL), | Seedling | FGOB10Ptm-1, PA14, CA17 (USA), DEN2.6 (Denmark), NZKF2 (New Zealand) | 2H | 14-40 | QRptm-2H-1-31 | Tamang et al 2019 | *SFNB-2H-8-10* |
| Pinnacle × **PI67381** (RIL), |  | DEN2.6 (Denmark) | 2H | 21 | QRptm-2H-77-83 |  | *Qns-2H.3* |
| Pinnacle × **PI84314** (RIL), |  | FGOB10Ptm-1 (USA) | 2H | 19 | QRptm-2H-126-137 |  | *QRptma2-3 ^#^* |
|  |  | FGOB10Ptm-1, PA14 (USA) | 2H | 17-25 | QRptm-2H-141-152 |  | *QRptma2-3 ^#^* |
|  |  | SG1 (Australia) | 3H | 20 | QRptm-3H-56-65 |  | *Rpt-3H-4 ^#^* |
|  |  | CA17 (USA) | 3H | 16 | QRptm-3H-81-88 |  | *QRpts3La* |
|  |  | DEN2.6 (Denmark), NZKF2 (New Zealand) | 4H | 26-34 | QRptm-4H-58-64 |  | *QPt.4H-3* |
|  |  | DEN2.6 (Denmark), NZKF2 (New Zealand) | 4H | 14=38 | QRptm-4H-120-125 |  | QRptm-4H-120-125 |
|  |  | FGOB10Ptm-1, PA14 (USA), DEN2.6 (Denmark) | 6H | 15-33 | QRptm-6H-55-64 |  | *AL_QRptt6-1* |
|  |  | DEN2.6 (Denmark) | 7H | 15 | QRptm-7H-92-95 |  | QRptm-7H-92-95 |
|  |  | FGOB10Ptm-1, PA14, CA17 (USA), SG1 (Australia) | 7H | 24-80 | QRptm-7H-119-137 |  | *QRptm7-6 ^#^* |
|  |  | FGOB10Ptm-1 (USA), SG1 (Australia), NZKF2 (New Zealand) | 7H | 15-28 | QRptm-7H-138-160 |  | *QTL_UHs_‐7H ^#^* |

* Resistant parental lines are indicated by bold type; DH, double haploid; RIL, recombinant inbred line; Artificial; breeding line panel; Natural; landraces and wild barley panel; Hybrid, artificial and natural barley panel.

† Stage at which resistance is active.

‡ County of origin given if isolate, location of infection given if natural infection

§ QTL effects containing ranges mean that the experiments were performed for multiple locations or multiple isolates and the effects for individual treatments fall into this range.

‖ References may contain more than one population.

¶ Inferred locus designation using respective references of colocalising loci using BarleyMap (Cantalapiedra et al., 2015) or T3/barley; earliest published designation given if locus was not previously designated; ^#^ not confirmed.

-, no information is available for this entry.

Updated and modified from Liu et al. (2011).
